# Soluble/MOF-Supported Palladium Single Atoms Catalyze the Ligand-, Additive-, and Solvent-Free Aerobic Oxidation of Benzyl Alcohols to Benzoic Acids

**DOI:** 10.1021/jacs.0c12367

**Published:** 2021-02-04

**Authors:** Estefanía Tiburcio, Rossella Greco, Marta Mon, Jordi Ballesteros-Soberanas, Jesús Ferrando-Soria, Miguel López-Haro, Juan Carlos Hernández-Garrido, Judit Oliver-Meseguer, Carlo Marini, Mercedes Boronat, Donatella Armentano, Antonio Leyva-Pérez, Emilio Pardo

**Affiliations:** † Instituto de Ciencia Molecular (ICMol), Universidad de Valencia, 46980 Paterna, Valencia, Spain; ‡ Instituto de Tecnología Química (UPV−CSIC), Universitat Politècnica de València−Consejo Superior de Investigaciones Científicas, Avda. de los Naranjos s/n, 46022 Valencia, Spain; § Departamento de Ciencia de los Materiales e Ingeniería Metalúrgica y Química Inorgánica, Facultad de Ciencias, Universidad de Cádiz, Campus Universitario de Puerto Real, 11510 Puerto Real, Cádiz, Spain; ∥ Instituto Universitario de Investigación en Microscopía Electrónica y Materiales (IMEYMAT), Facultad de Ciencias, Universidad de Cádiz, Campus Universitario de Puerto Real, 11510 Puerto Real, Cádiz, Spain; ⊥ CELLS−ALBA Synchrotron, Cerdanyola del Vallès, E-08290 Barcelona, Spain; # Dipartimento di Chimica e Tecnologie Chimiche (CTC), Università della Calabria, Rende 87036, Cosenza, Italy

## Abstract

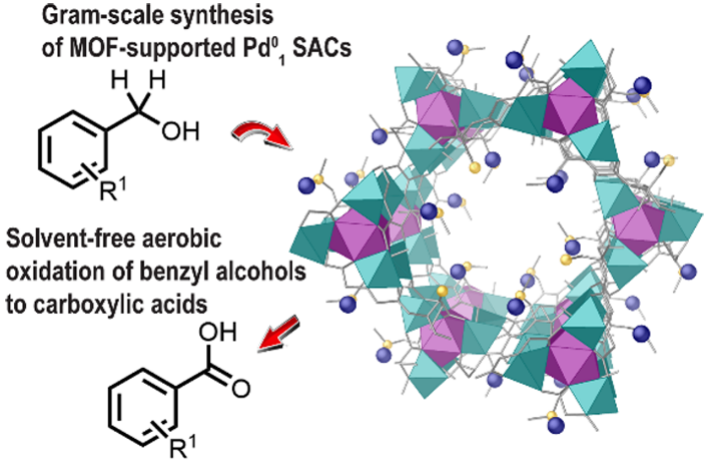

Metal single-atom catalysts (SACs) promise great rewards in terms of metal atom efficiency. However, the requirement of particular conditions and supports for their synthesis, together with the need of solvents and additives for catalytic implementation, often precludes their use under industrially viable conditions. Here, we show that palladium single atoms are spontaneously formed after dissolving tiny amounts of palladium salts in neat benzyl alcohols, to catalyze their direct aerobic oxidation to benzoic acids without ligands, additives, or solvents. With this result in hand, the gram-scale preparation and stabilization of Pd SACs within the functional channels of a novel methyl-cysteine-based metal–organic framework (MOF) was accomplished, to give a robust and crystalline solid catalyst fully characterized with the help of single-crystal X-ray diffraction (SCXRD). These results illustrate the advantages of metal speciation in ligand-free homogeneous organic reactions and the translation into solid catalysts for potential industrial implementation.

## Introduction

Single-atom catalysts (SACs) attract great interest due to their unique catalytic properties in different reactions of capital importance.^1^ However, various limitations linked with their real applications, such as the difficulties related to their gram-scale preparation, their challenging characterization, and the need to use protective ligands to stabilize these SACs, preventing their agglomeration, still need to be overcome.^2−4^ In this context, the formation, stabilization, and catalytic action of soluble SACs in the neat reactant is still a quite unexplored field, since metals, even in very low amounts, tend to agglomerate in organic solutions after reduction.^5−7^ However, this may not be the case for molecules able to concomitantly reduce, stabilize and be activated by the *in situ* formed SACs.^8^

**Figure 1:**
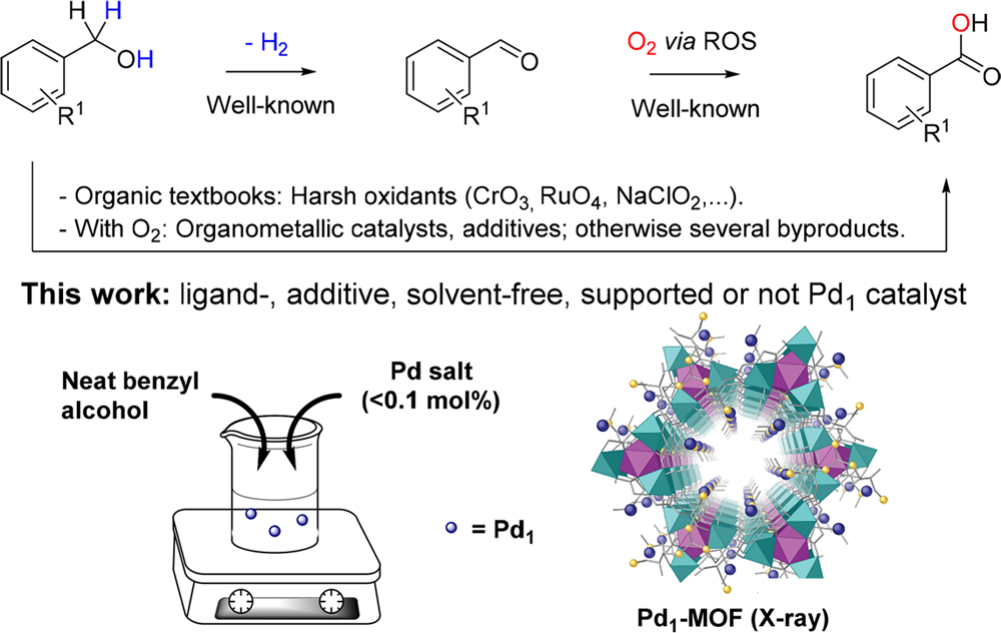
Stepwise and direct catalytic oxidation of benzyl alcohols to benzoic acids (top) and the catalytic systems used here (bottom).

Benzyl alcohols are fundamental starting materials in organic synthesis such as in the formation of benzaldehydes and benzoic acids after oxidation.^9,10^Figure 1 shows that the dehydrogenation of benzyl alcohols occurs under a variety of metal catalysts and reaction conditions^11−16^ and that the radical oxidation of benzaldehydes occurs spontaneously under air for substrates with >98% purity; otherwise, just a >2% of remaining benzyl alcohol acts as a very good quencher of radical oxygen species (ROS).^17^ With these results in mind, it is not surprising that in contrast to the stepwise process the direct aerobic oxidation of benzyl alcohols to benzoic acids is a challenging reaction that requires of harsh oxidation agents or organometallic complex catalysts, additives, and solvents; otherwise, different undesired reactions such as ether and ester formation occurs.^18−28^

Benzyl alcohol is an industrial reagent for the reduction of noble metal salts (e.g., Pd, Au, etc.) to nanoparticles, with the concomitant formation of benzaldehyde.^29^ Thus, we envisioned that perhaps the dissolution of a metal salt into tiny amounts of neat benzyl alcohols could generate a viable, self-stabilized redox single metal atom for the catalytic oxidation of benzyl alcohols to benzoic acid under air. This highly reactive single atom would, in principle, feature the empty coordinating sites required for the different chemical events during reaction, including dehydrogenation and oxygen activation,^19,20^ while most likely circumventing severe benzyl alcohol poisoning. From a material synthesis point of view, the SAC could be considered an arrested state of the metal during the reduction/aggregation process into the benzyl alcohol, which keeps the metal seeds alive and catalytically active for the solvent-, ligand-, and additive-free oxidation reaction, beyond which some other metal species could be present.^30−33^

## Results and Discussion

**Figure 2:**
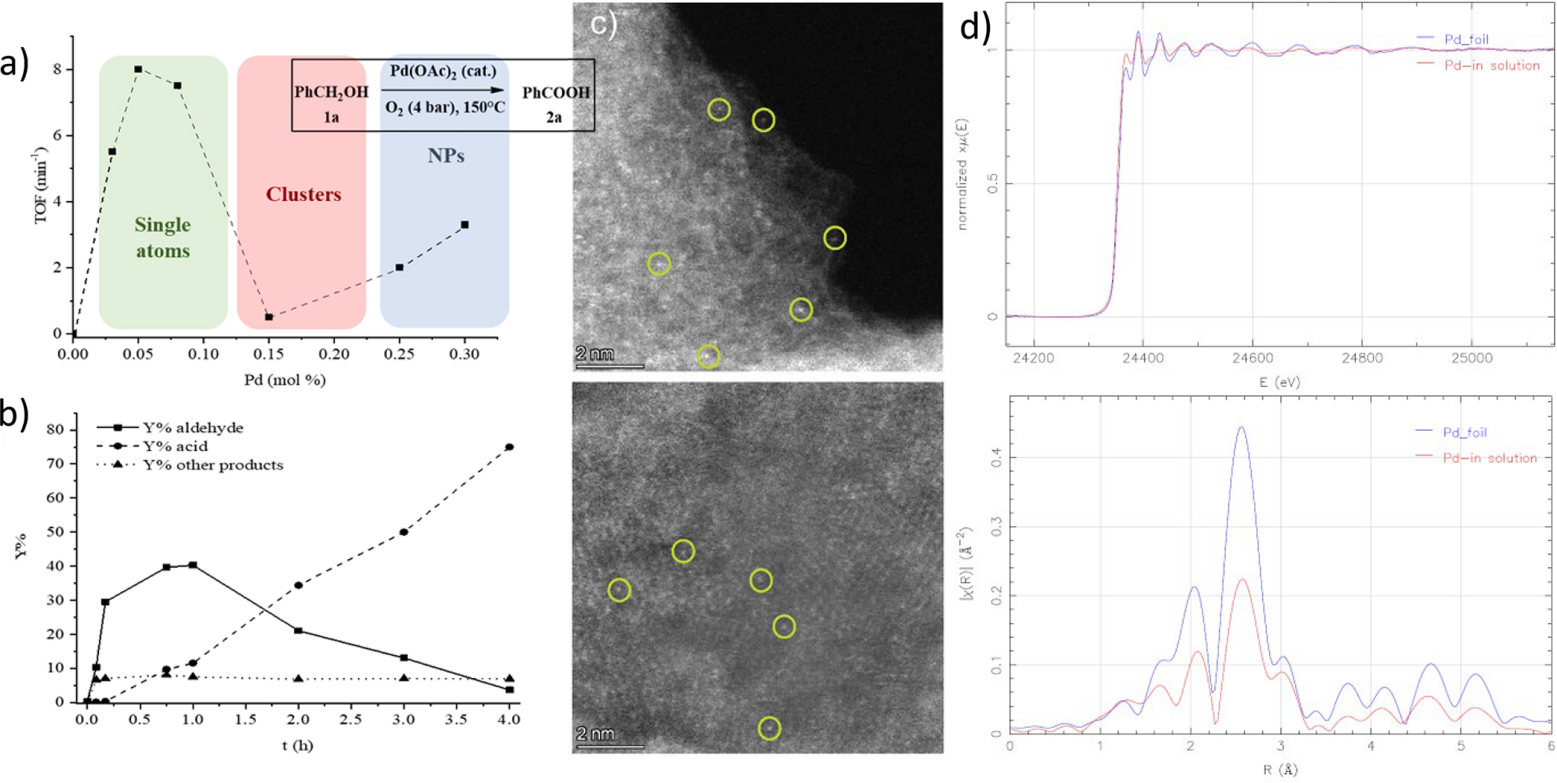
(a) Initial turnover frequency (TOF_0_) for the aerobic oxidation of neat benzyl alcohol **1a** with increasing amounts of Pd(OAc)_2_ at 150 °C under 4 bar of O_2_. (b) Representative time–yield kinetic plot of the reaction for 0.05 mol % Pd(OAc)_2_. (c) Two different AC-HAADF-STEM images of the Pd species in solution during reaction, after being trapped in active charcoal. Some Pd SACs are marked with yellow circles. (d) X-ray absorption near-edge structure (XANES, top) and extended X-ray absorption fine structure (EXAFS, bottom) spectra of the solution (red lines), compared to Pd foil (blue lines).

Figure 2a shows the catalytic results for the aerobic oxidation of neat benzyl alcohol **1a** to benzoic acid **2a** with a 0.03–0.3 mol % of dissolved Pd(OAc)_2_, and it can be observed that a high initial turnover frequency (TOF_0_) for **2a** is achieved with <0.1 Pd mol % but not with higher amounts of Pd, with yields of **2a** around 50–80% after 4 h of reaction time (Figure S1). Similar results were observed with other Pd sources, including K_2_PdCl_4_, Pd_2_(dba)_3_, and Pd(acac)_2_, but not with Pd complexes having a stronger ligand such as a phosphine, i.e., Pd(PPh_3_)_4_ or Pd(PPh_3_)_2_Cl_2_ (Table S1 and Figure S2). Figure 2b shows that remarkably **2a** starts to form at intermediate conversions, when >50% of **1a** still remains in solution. These results confirm that the Pd catalyst formed under these conditions is able to override the poisoning of **1a** under aerobic conditions. An acceptorless dehydrogenation pathway can be ruled out since an open vial reaction gave very conversion of **1a**, thus confirming the need of O_2_ to facilitate the one-pot oxidation.

Figure 2c shows aberration-corrected high-angle annular dark field scanning-transmission electron microscopy (AC-HAADF-STEM) measurements of the metal species in solution during reaction, trapped *in situ* with active charcoal. Since AC HAADF-STEM imaging is proportional in good approximation to the squared atomic number, Z^2^, Pd species can be reliably identified as the brightest contrasts in the image (some of them have been marked with orange circles). While Pd single atoms are the main species present at <0.1 Pd mol %, only clusters and eventually NPs were found at >0.1 Pd mol (see Figures S3 and S4). Figure 2d shows X-ray absorption near edge structure (XANES) and extended X-ray absorption fine structure (EXAFS) measurements of the solution, which confirm the reduction of Pd and the generation of very small agglomerates, with an average number of ∼6 Pd–Pd bonds (see also Table S2), much lower than in Pd foil (12 Pd–Pd bonds). Ultraviolet–visible (UV–vis) spectrophotometric titrations with PPh_3_ confirm the progressive disappearance of Pd^2+^ in solution during reaction (Figure S5 top). These results strongly support that partially reduced Pd1 species could be the catalytic active species for the direct aerobic oxidation of **1a** to **2a**.

The very low catalytic activity found with intermediate Pd amounts (0.1–0.25 mol %) is consistent with the formation of subnanometric Pd clusters, catalytically inactive in this case.^6^ In order to check this hypothesis, subnanometric Pd clusters in solution were independently prepared by two reported methods, i.e., endogenous reduction in aqueous *N*,*N*-dimethylformamide^6^ and supporting solvent as well as reduction and leaching from ethylene vinyl alcohol polymer (EVOH),^8^ and tested as catalyst for the oxidation of **1a** under the same conditions than Pd salts and complexes. The results (Figure S5, bottom) show that these clusters are inactive as catalysts for the oxidation of **1a** to **2a**, which strongly supports that Pd_1_ is the main catalytic active species for the oxidation reaction. It is reasonable to think that the combination of a mild reductant agent (benzyl alcohol), which can at the same time act as a stabilizer, could have the same effect than a support/strong reductant system for the preferential formation of Pd_1_ species.^1−3^ In any case, the Pd clusters may be unable not only to catalyze the redox reaction but also to dislodge Pd single atoms, according to the canonical Ostwald ripening mechanism. In accordance with this hypothesis, the formation of **2a** starts to be observed again at Pd concentrations where NPs are formed (>0.3 mol %). This is in good agreement with the reported catalytic activity of some metal NPs for this reaction,^23−28^ as well as with the ability of metal NPs to dissociate O_2_ and dislodge single atoms in solution.^34^ Commercially available samples of Pd/C with different Pd loadings (1–10 wt %) and particle size (5–50 nm average diameter) were tested as catalyst for the reaction and only the sample with highest loading and biggest NP size was active for the formation of **2a** (Figure S6). A quenching test with triphenylphosphine, under the indicated reaction conditions, showed that the catalytic activity comes from species in solution (Figure S7). These results strongly support that the oxidation of **1a** to **2a** in the neat reagent is catalyzed by soluble Pd1 species, regardless the amount of Pd employed.^35^

SACs are, by definition, supported metal species.^1−4^ Thus, once we established the catalytic activity of *in situ* prepared Pd_1_ SACs for the one-pot oxidation of **1a** to **2a**, it is of interest to find a solid able to generate and stabilize such Pd^0^_1_ species. However, this is a quite difficult task. The appropriate solid has to be able to preserve the required electronic and structural chemical nature of SACs, preventing their leaching out under reaction conditions and diffusing onto the solid to aggregate and, at the same time, enable a clear-cut characterization of the supported Pd_1_ site and surroundings. In this context, metal–organic frameworks^36−41^ (MOFs) are one of the most suitable platforms to overcome these difficulties. MOFs chemistry have reached high microporosity control, fine-tuning of the functionalities decorating their channels, and in-depth characterization of the final hosted metal species using single-crystal X-ray diffraction (SC-XRD).^42−48^ As a direct consequence, MOFs have experienced rapid growth over the few last decades in catalysis, by means of their constituting building blocks, both organic linkers and open metal sites, and/or catalytically active guest species within their pores.^49−57^

**Figure 3:**
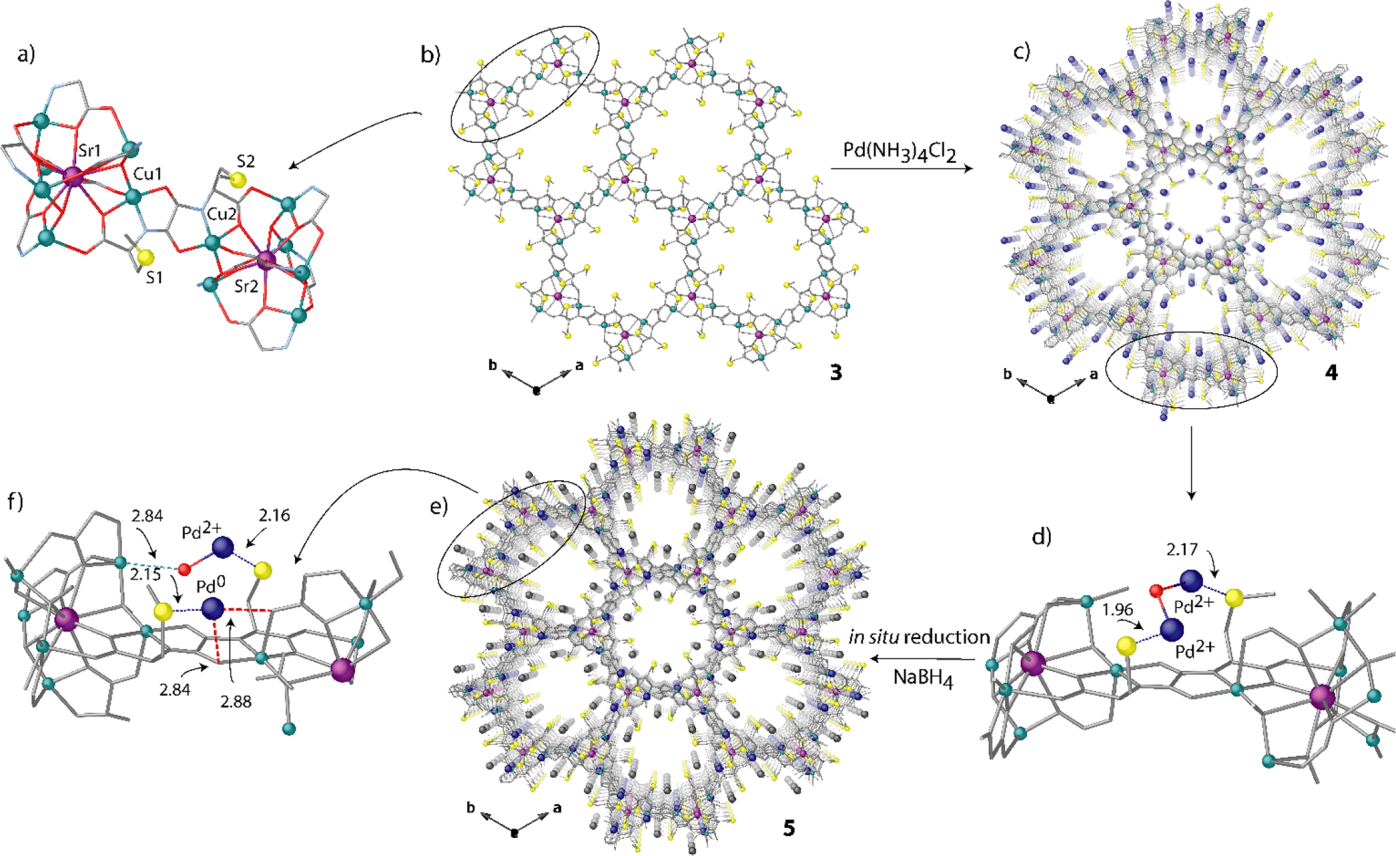
PS approach showing the structures of **3**–**5** determined by single-crystal X-ray diffraction which consists of two consecutive processes: first, the insertion of [Pd(NH_3_)_4_]^2+^ cations within the channels of **3** (a, b) to give **4** (c, d) and, second, the reduction of Pd^2+^ cations to form the Pd^0^_1_ single atoms in **5** (e, f). Copper and strontium atoms from the network are represented by cyan and purple spheres, respectively, whereas organic ligands are depicted as gray sticks. Yellow and blue spheres represent S and Pd atoms (gray spheres in e represent Pd^0^ atoms). Dotted lines represent the Pd···S interactions.

Herein, we report a novel three-dimensional (3D) MOF, derived from the amino acid *S*-methyl-l-cysteine, with formula {Cu_6_Sr[(*S*,*S*)-Mecysmox]_3_(OH)_2_(H_2_O)}·15H_2_O (**3**) (Mecysmox = bis[*S*-methylcysteine]oxalyl diamide) (Figure 3a), featuring pores densely decorated with dimethyl thioether groups, which allow the sequential formation and stabilization of Pd_1_ SACs within their functional channels (Figure 3). In order to do so, a two-step postsynthetic (PS)^57^ strategy has been applied, leading to the formation of two novel adsorbates with formulas [Pd^II^_2_(H_2_O)(NH_3_)_6_]_0.5_Cl_2_@{Sr^II^Cu^II^_6_[(*S*,*S*)-Mecysmox]_3_(OH)_2_ CH_3_OH)}}·12H_2_O (**4**) (Figure 3c) and (Pd^0^_1_)_0.5_([Pd^II^(H_2_O)(NH_3_)_3_]Cl_2_)_0.5_@{Sr^II^Cu^II^_6_[(*S*,*S*)-Mecysmox]_3_(OH)_2_ CH_3_OH)}}·13H_2_O (**5**), respectively, (Figures 1 bottom and 3e, Table S3). Figure 3 serves a dual purpose: shows the crystal structures of **3**–**5** and illustrates the SACs formation route. First, this consists of the insertion of [Pd(NH_3_)_4_]Cl_2_ cations in the starting MOF (**3**) to form the Pd^2+^-containing MOF (**4**) and the concomitant *in situ* reduction of half of the Pd^2+^ cations to form the mixed valence Pd^2+^/Pd_1_ hybrid compound (**5**) (see the Experimental Section). Notably, the sulfur-containing groups play a dual key role in this PS approach. They retain the Pd^2+^ cations in specific positions after the insertion process, which allows their homogeneous distribution along the channels and prevents their agglomeration and forming of NCs or NPs during the reduction process. Besides, the crystal structure of each phase could be unveiled by SC-XRD given the high crystallinity and robustness^58−64^ of the pristine MOF (Figure 3 and Table S4).

**Figure 4:**
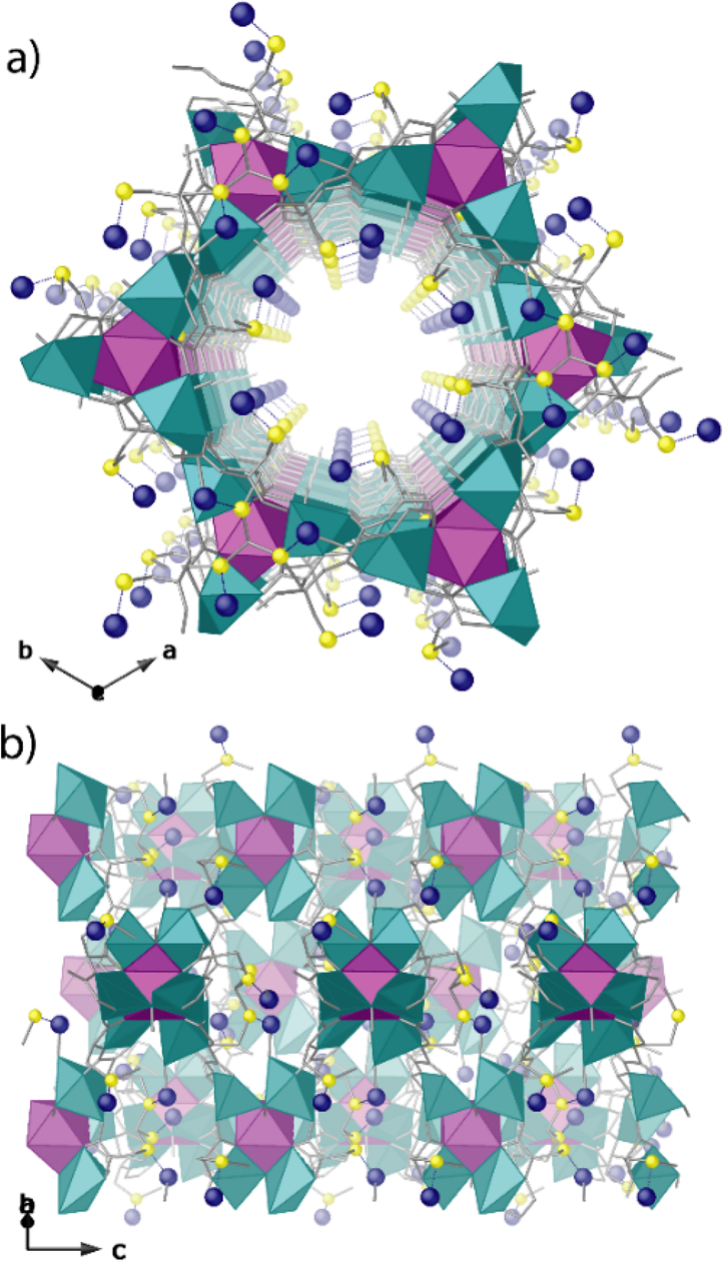
Perspective views of one single channel of **5** along the *c*- (a) and *b*-axes (b). Copper and strontium atoms from the network are represented by cyan and purple polyhedra, respectively, whereas organic ligands are depicted as gray sticks. Yellow and blue spheres represent S and Pd atoms. Dual color sticks represent the Pd···S interactions.

Compounds **3**–**5** are isomorphous, crystallizing in the chiral *P*6_3_ space group, and exhibit a chiral 3D strontium(II)–copper(II) network, featuring hexagonal channels where the dimethyl thioether chains from methylcysteine residues are confined. These functional arms exploit their intrinsic flexibility adopting different most stable conformations depending on the nature of target, i.e., solvent molecules in **3** (Figures S8–S10), Pd^2+^ in **4** (Figures 3c,d and S11) or both Pd^2+^ cations and Pd^0^_1_ SACs in **5** (Figures 3e,f, 4, and S14). The Pd^2+^–S bond distances [1.96(4) and 2.17(2) Å (4)] (Figures 3d and S13a) are close enough to those observed previously,^65^ and similar to the Pd^0^–S bond distance observed in **5** 2.16(2) Å] (Figures 3f and S13b). In **3**–**5** the thioether chains from the Mecysmox ligand show as basic conformation one of the two distinct moieties in a distended conformation toward the center of the pores, and the other one regularly bent, with the terminal methyl groups pointing toward the smaller interstitial voids residing along the *a*-axis (Figures S10 and S14). Both conformations allow amino acid arms to efficiently target Pd^2+^ ions by S binding sites,^48,66−68^ as confirmed by the crystal structure of **4**. However, only the Pd^2+^ ions residing in the most accessible pores [50% of total Pd^2+^ ions] could be chemically reduced to Pd^0^, as confirmed by XPS spectrum of **5** (vide infra) (Figures 4b and S11–S14). This situation was observed previously.^62^ In **5**, Pd_1_ SACs are fixed to sulfur atoms in the larger hexagonal pores (Figure 4a and S12), whereas Pd^2+^ ions are still stabilized by sulfur atoms of dimethyl thioether located in interstitial voids (Figure S14). The water molecule acting as bridge between two-coordinated Pd^2+^ ions in **4**, still remains coordinated in **5** but as a terminal ligand, to only one of Pd^2+^ metal ions, as a consequence of the breaking linkage after Pd_1_ SAC formation [Pd–Ow 2.00(6) and 3.03(7) Å in **4** and 1.99(2) Å in **5**] (Figure S13) (vide infra). Pd^0^_1_ weakly also interacts with oxamate moieties with Pt···O distances of 2.84(1) and 2.88(1) Å (see also Figures S12 and S13b). As far as we know, no examples of crystallographically precise Pd_1_ SACs have been reported so far. Nevertheless, this Pd···O distance is close enough to that previously reported for Pd nanoclusters (2.9 Å).^64^

SC-XRD data also allows to suggest certain parameters of the Pd_1_ formation mechanism. First, the bridging water molecule in [Pd_2_(H_2_O)(NH_3_)_6_] units (**4**) (Figures 3d and S13a) might play a crucial role during the SACs formation process (**5**), acting synergistically with the flexible dimethyl thioether chains from methylcysteine residues stabilizing Pd^2+^ metal ions in **4**. The reduction process of Pd^2+^–S units located inside the most accessible pores breaks water bonds from one side, which meanwhile generates the Pd^0^–S ones in **5**. This leaves the water molecule still coordinated and stabilizing the unreduced mononuclear Pd^2+^ complexes residing in hindered interstitial voids (Figures 3f and S13b). Second, the length of the amino acid residue also seems to play a key role in the nuclearity of the metal species formed. Thus, in the present MOF, where Pd^2+^ cations are connected to shorter dimethyl thioether chains within the channels, Pd_1_ SACs are formed during the reduction process. In turn, in a previously reported work with an isoreticular MOF prepared from the amino acid l-methionine,^66,67^ the larger length of the ethylmethyl thioether chains decorating the channels allows a closer approach of the metal species, and dinuclear Pt_2_ nanoclusters could be obtained.^62^

The virtual diameter of the channels only slightly decreases from ca. 0.9 nm in the precursor material **3** to ca. 0.7 nm in **4** and **5** (Figures S9, S11, and S12). This is in total agreement with adsorption measurements. Thus, the permanent porosity of the samples, particularly important in the case of **4** and **5** for catalytic applications, was verified by measuring their N_2_ adsorption isotherms at 77 K. They confirm the permanent porosity for **3**–**5** (Figure S15), which is slightly lower for **4** and **5**, as expected from the decrease of their accessible void due to the presence of the Pd guests within the channels (calculated Brunauer–Emmett–Teller (BET) surface areas^69^ for **3**, **4**, and **5** are 719, 548, and 572 m^2^/g, respectively).

Besides the structural characterization, the chemical identities of **3**–**5** were also established by elemental analyses (C, H, S, and N), inductively coupled plasm–mass spectrometry (ICP–MS), powder X–ray diffraction (PXRD), electronic microscopy, X-ray photoelectron spectroscopy (XPS) and thermogravimetric (TGA) analyses (see the Supporting Information). Figure S16 shows the experimental powder X-ray diffraction (PXRD) patterns of **3**–**5**. They are identical to the theoretical ones (bold lines in Figure S16), which confirms that the bulk samples are pure and homogeneous. The solvent contents of **3**–**5** were, however, definitively established by TGA (see Figure S17). The XPS spectra of **4** and **5** are shown in Figure S18. The Pd 3d line of **2** is only one doublet with a binding energy (BE) of the Pd 3d_5/2_ peak of 337.8 eV, typical of Pd^2+^ cations (Figure S18a) which is close enough to other reported values.^64^ In turn, Figure S18b clearly shows, apart from the same Pd 3d_5/2_ doublet with a BE of 337.7 eV, an additional peak at 335.8 eV, attributed to reduced Pd^0^_1_ SACs,^64^ with a 1:1 ratio respect to Pd^2+^. This feature indicates that only 50% of Pd^2+^, those occupying accessible positions, are reduced when in contact with reducing agent, whereas those Pd^2+^ cations situated in inaccessible sheltered interstitial positions (see structural description) remain in their original oxidation state. These values are close enough to those observed for other Pd^2+^/ Pd_1_ species.^64^ Therefore, they suggest that the thioether groups do not alter significantly either the native electronics nor the open-shell structure of the Pd_1_ site, which is ready to catalyze the aerobic oxidation of **1** to **2**.

**Figure 5:**
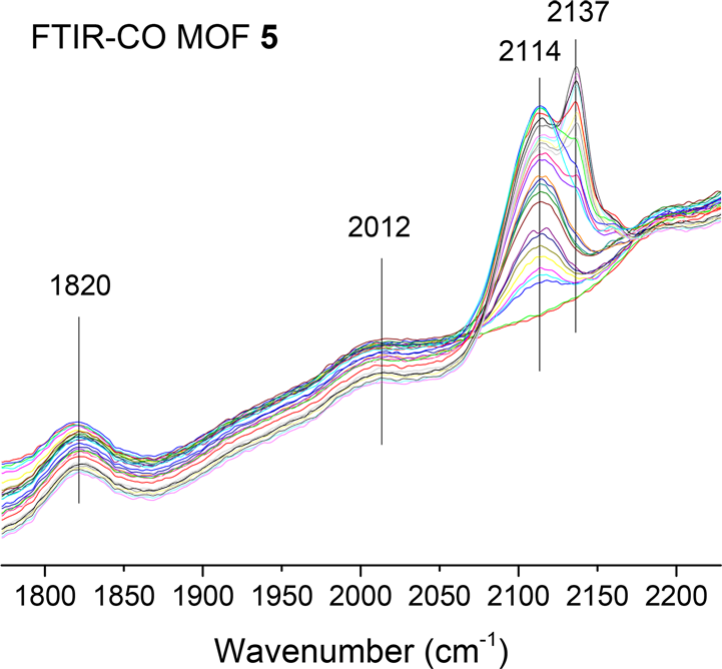
Low-temperature Fourier transform infrared spectrum, under CO (FTIR-CO), of fresh MOF **5**. Peak assignments: free CO (2137 cm^–1^), Pd^2+^ (2114 cm^–1^), Pd^δ+^ (2012 cm^–1^, δ = 0–1), and Pd^0^ NPs (1820 cm^–1^).

In order to further confirm the presence of partially reduced Pd SACs within **5**, Fourier transform infrared under CO (FTIR–CO), XANES, and EXAFS spectroscopic measurements and computational calculations based on the density functional theory (DFT) were carried out. Figure 5 shows the low-temperature (−196 °C) FTIR-CO results of **5**, where there are no signals above 2150 cm^–1^, corresponding to bare Pd^2+^, can be observed, which supports the partial reduction of Pd. However, two clear broad signals centered at 2114 and 2012 cm^–1^, attributable to unreduced Pd^2+^ and highly dispersed, partially reduced Pd^δ+^ atoms (δ = 0–1),^64^ respectively, can be clearly seen, together with the increasing sharp signal of free CO (2137 cm^–1^) at high CO doses. These peaks are accompanied by a very broad signal at 1820 cm^–1^, which can be assigned to Pd(0) nanoparticles.^64^

**Figure 6:**
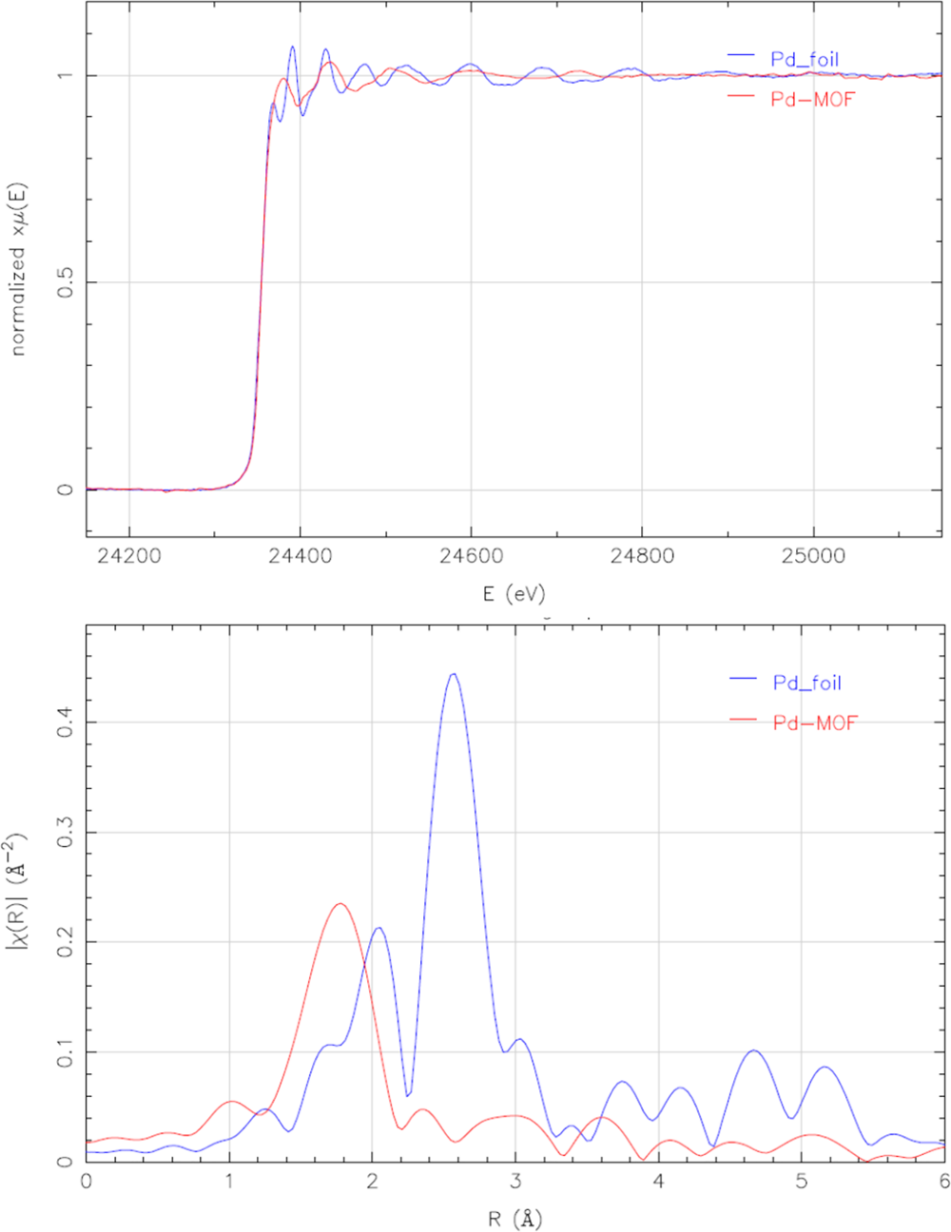
XANES (top) and EXAFS (bottom) spectra of fresh MOF **5** (red lines), compared to Pd foil (blue lines).

Figure 6 shows the EXAFS and XANES spectra of MOF **5**, compared to Pd foil. The results confirm the partial reduction of Pd, as occurred for the Pd catalyst in solution (see Figure 2d above and Figure S19 for comparison and fitting). It can be observed in both cases, i.e., in solution and in MOF **5**, that the first oscillations beyond the edge are flattened respect to the foil due to quantum size effects of the single atoms, also indicating a large fraction of low coordination Pd atoms, more intensified in the case of MOF **5**. No Pd–Pd bond signals can be detected for the latter, but an average of 3 Pd–S bonds are detected (see also Table S2 and the Experimental Section in the Supporting Information, with references), in nice agreement with the SCXRD structure. Combined, these results strongly support the single-atom nature of Pd within MOF **5**.

**Figure 7:**
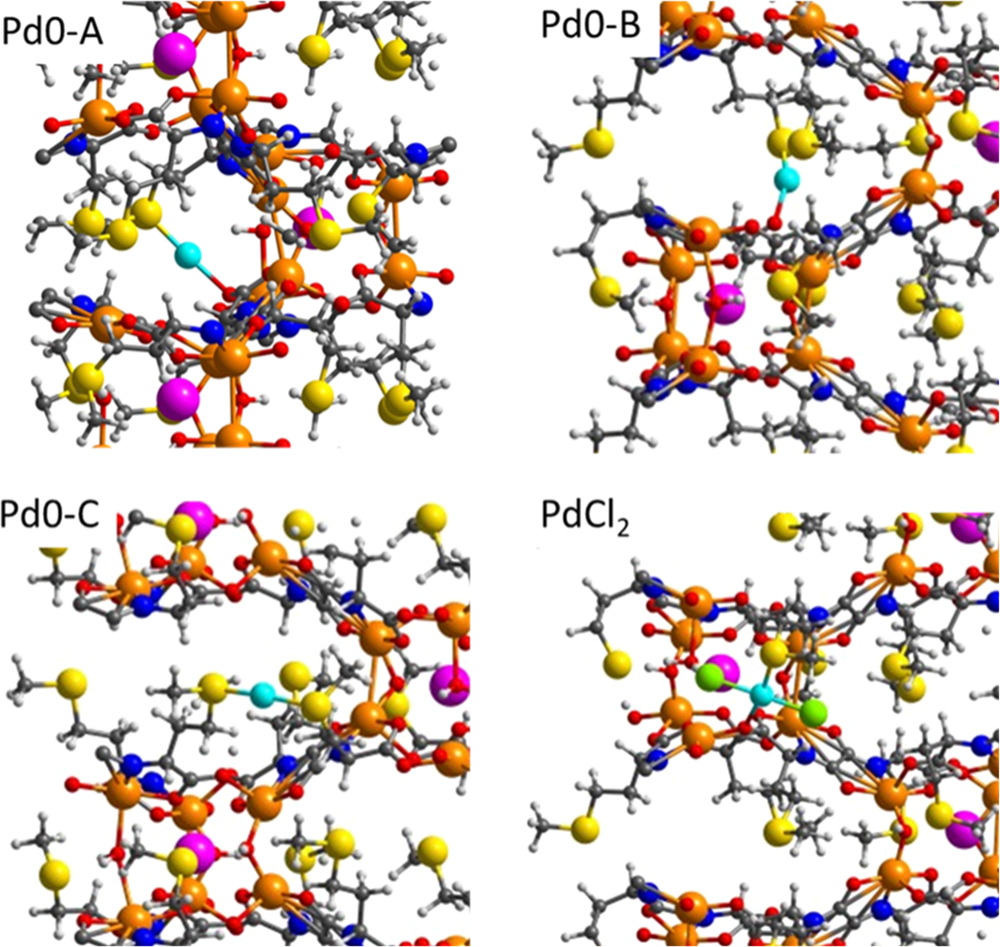
DFT-optimized structures of Pd(0) and PdCl_2_ within the MOF, for three different coordination environments (Pd0–A–C, see also Figure S20 and Table S5). Pd, Cu, Sr, S, N, O, Cl, C, and H atoms are depicted as cyan, orange, pink, yellow, blue, red, green, gray, and white balls, respectively.

In order to further support the stability of the Pd SACs in MOF **5**, DFT calculations were performed. Figure 7 shows the periodic DFT calculations through geometry optimization of Pd(0) in three different environments within the MOF (see also Figure S20 and Table S5), which support the crystallographic characterization of **5**. Pd(0) is always linearly coordinated to two ligands (O or S) with optimized Pd–O and Pd–S distances between 2.1 and 2.3 Å, as measured in the X-ray absorption spectroscopy (XAS) techniques (compare with Table S2). The calculated atomic charge on Pd in PdCl_2_ within the MOF is nearly the same as in the gas phase calculated at the same level of theory (0.678 e), while the net atomic charges on two Pd(0) models are just slightly positive and slightly negative in the third one (see Table S5). With the optimized structure of MOF **5** in hand, the interaction of the Pd SACs with CO was simulated by DFT calculations, in order to compare with the experimental results in Figure 5. The results (Table S6 and Figure S21) show that CO interacts strongly with all models of Pd0 but not with PdCl_2_, with calculated interaction energies between −28 and −40 kcal·mol^–1^ and with optimized Pd–CO distances around 1.8 Å. In particular, the interaction of Pd with CO in the Pd0–A system is so strong that the Pd–O bond is broken. The calculated ν(CO) frequency in this system, 1997 cm^–1^, is similar to that obtained for CO adsorbed on top of a corner Pd atom in a small Pd_13_ cluster used as reference (1999 cm^–1^) and nicely fits with the observed experimental peak at 2012 cm^–1^ (see above). In the other two models (Pd0–B–C), where the Pd(0) atom remains attached to two MOF ligands after CO coordination, the calculated ν(CO) frequency is slightly shifted to 1930–1945 cm^–1^, in any case still assignable to the experimental peak observed.

**Figure 8:**
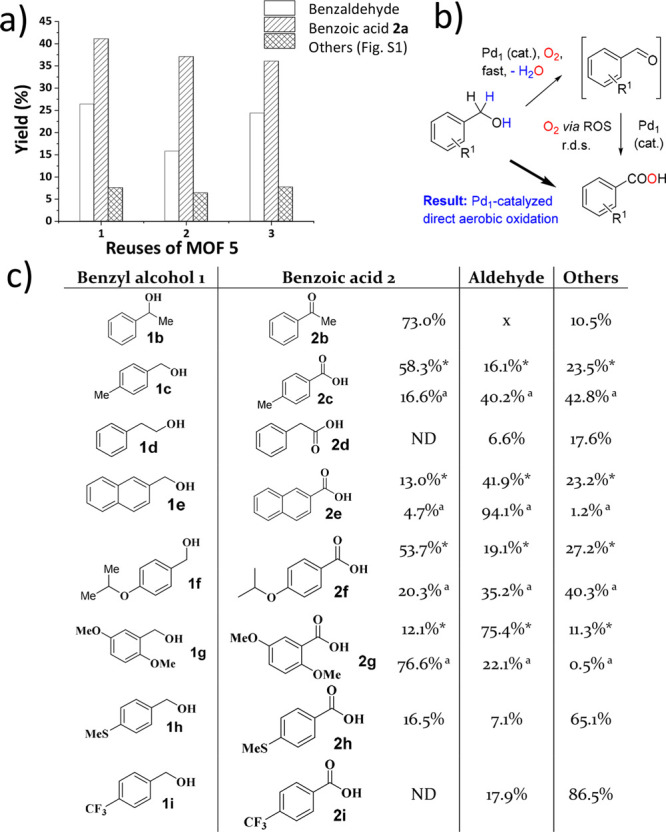
(a) Reusability of Pd^0^_1_ SACs-MOF (**5**); reaction conditions: 1.96 mmol substrate, 0.1% mol Pd^0^_1_ SACs-MOF, 4 atm O_2_, 150 °C, 450 rpm, 15 h; GC yields. (b) Plausible reaction mechanism for Pd in solution. (c) Reaction scope with Pd(OAc)_2_ (0.3 mol %). Reaction conditions: 1.96 mmol substrate, 0.3% mol Pd(OAc)_2_, 4 atm O_2_, 150 °C, 450 rpm, 4 h. GC yields. *15 h. ^*a*^0.1% mol Pd^0^_1_ SACs-MOF, 24 h.

**Figure 9:**
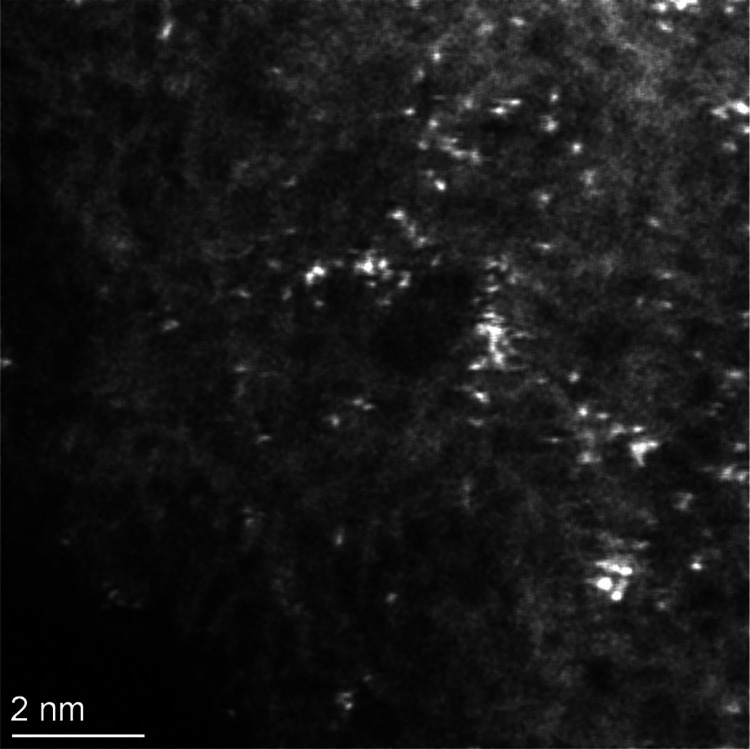
AC-HAADF-STEM image of reused MOF **5** showing the presence of Pd SACs.

The catalytic results for the aerobic oxidation of benzyl alcohol **1a** with MOFs **3**–**5** show that only Pd_1_ SACs-MOF (**5**) catalyzes the oxidation with good efficiency (28% of benzaldehyde and 43% of benzoic acid **2a**). In contrast, **4** barely catalyzes the reaction, and **3** is completely inactive. The inactivity of **4** can be explained by the need of using a reducing agent stronger than **1a** to obtain the catalytically active Pd_1_ species within the MOF, which then show a catalytic activity comparable to the Pd(0) complex Pd_2_(dba)_3_ (see Table S1). Figure 8a shows that MOF **5** is recyclable, without minimal depletion of the catalytic activity after 3 reuses. In order to verify the integrity of the SACs in MOF **5**, electronic microscopy experiments were carried out after the catalytic experiments, where no SC-XRD measurements could be carried out due to the loss of the crystallinity of the material. Figures 9 and S22 show representative AC-HAADF-STEM images of **5**. Highly dispersed Pd species are clearly observed. In particular, they show a 0.135 nm average diameter, which is in a good agreement with isolated atomic species. Only very scarce, small agglomerations could be observed in some areas, with diameters <0.5 nm, thus confirming that the Pd atoms do not aggregate into large NCs. In the same vein, PXRD pattern of **5**, recovered after catalysis (Figure S23a), confirm that the material remains crystalline and that no characteristic XRD peaks of Pd NPs or oxides are observed, further confirming the integrity of Pd_1_ SACs. Moreover, the XPS spectra of **5** after catalytic experiments (**5′**) is very similar to that of the starting material (Figure S23b), confirming that a 1:1 ratio for Pd^0^ and Pd^2+^ remains after catalysis. In accordance with all the characterization made to the used MOF **5** sample, leaching tests after filtration in hot of the catalyst (Figure S24) reveals that no reaction occurs after filtration of the solid catalyst, neither for the benzaldehyde intermediate nor product **2a**, which disproves the presence of catalytically active Pd species in solution from MOF **5**.

The potential cocatalysis by the Cu atoms in the MOF was discarded on the basis of comparative experiments (Table S7), since Cu(OAc)_2_ merely does not catalyze the reaction (0.6% of **2a** and 2.8% of benzaldehyde under optimized reaction conditions), while a Cu-MOF treated under reduction conditions (NaBH_4_ in methanol) and not having any Pd showed a similar catalytic activity than Cu(OAc)_2_ (0.8% of **2a** and 5.0% of benzaldehyde). These results disprove Cu, on its own, as a catalyst of the reaction, and when Cu(OAc)_2_ was put together with Pd(OAc)_2_, the yield of benzoic acid **2a** was lower than that with Pd alone. These results together confirm that Pd is the only metal catalyst for the reaction here. Overall, these results nicely fit the observations during the reactions in solution and strongly support the idea that ligand-free Pd_1_ are the catalytically active species during the one-pot oxidation of benzyl alcohols to benzoic acids under additive- and solvent-free conditions.

Kinetic experiments evidence that the rate equation for Pd_1_ in solution is *v*_0_ = *k*_exp_[Pd][O_2_][**1a**]^−1^ (Figure S25); this equation rate is similar if one starts from benzaldehyde rather than benzyl alcohol **1a** (Figure S26) and that the kinetic isotopic effect (KIE) is 2.6(7) when **1a-*****d*****_2_** is used as the neat substrate. The inverse reaction order for **1a**, obtained by dilution experiments with *n*-hexadecane, is in accordance with the expected tendency of **1a** to poison the oxidation catalyst. The good linearity of the reaction rate with O_2_ pressure supports the lack of diffusion effects and sufficient solubility in the neat reactant.^70^ Trapping of benzaldehyde as an acetal with a diol, *in situ*, completely stops the formation of benzoic acid **2a**, and starting the reaction from the corresponding ester does not give any product **2a**, while dibenzyl ether does give **2a** (Figures S27 and S28). Moreover, the addition of the oxygen radical inhibitor DABCO to the reaction mixture stops the formation of **2a**, but it did not affect the formation of benzaldehyde (Figure S29). These kinetic, isotopic, and reactivity results together strongly support the reaction mechanism proposed in Figure 8b for Pd in solution, where the rate-determining step of the reaction is the oxidation of benzaldehyde to **2a**. This explains the catalytic activity of Pd_1_ in the neat reactant, since the dehydrogenation of **1a** proceeds extremely well, and no base or additional stabilizing are needed. However, given that this is an early step in the reactive sequence, the addition of catalytic amounts of NaOAc did increase the reaction rate (Figure S30).^18−28^ In contrast, the rate equation obtained with MOF **5** as a catalyst on the basis of initial rates (Figure S31) is *v*_0_ = *k*′_exp_[**5**][**1a**], which differs from that of soluble Pd (compare with Figure S25). The lack of influence of O_2_ for the solid catalyst can be explained by diffusion limitations; thus, the dehydrogenation step is slower than that in solution. Indeed, the experimental activation energy for the latter (7.7 kcal·mol^–1^), also on the basis of initial rates, is much higher than that for the former (35.2 kcal·mol^–1^), showcasing the more difficult access to the catalytic sites in the solid. The inhibiting effect with the strong ligand PPh_3_ also occurs (Table S1).

The fact that different Pd sources work well as catalysts in solution (Table S1) suggests that the dynamic system drives to a common catalytically active reduced Pd species in variable amounts, while in contrast the reduced Pd species are directly obtained within the MOF **5** during the reduction treatment and not during reaction, since MOF **4** does not work well. These results illustrate the stability conferred by the MOF structure to the confined Pd single atoms, at expense of losing substrate availability. Nevertheless, Figure 8c shows the reaction scope for the Pd_1_ catalyst in neat benzyl alcohols **1a**–**i**, which provides a limited number of benzoic acids **2a**–**i** in moderate yields, fairly comparable to most of the catalytic metal systems previously reported.^71^ Besides, the calculated turnover frequency for product **2a** under optimized conditions is 7.95 min^–1^, which is a 50-fold increase with respect to any other catalytic system previously reported for this reaction (Table S8).

## Conclusions

In summary, we report, in the first part of the manuscript, the *in situ* formation of Pd_1_ in neat benzyl alcohols which are able to catalyze the aerobic oxidation to benzoic acids. Then, we present the gram-scale preparation of well-defined Pd_1_ SACs in a methyl-cysteine-based MOF, homogeneously distributed and stabilized along the functional channels. Synchrotron SC-XRD allows, for the first time, to clearly visualize the Pd_1_ SACs and surroundings. The nature of Pd_1_ in both solution and MOF is further supported by microscopic and XAS techniques, in addition to DFT calculations for the solid. The latter enable us to support SC-XRD results unveiling the main interactions between palladium atoms and the network, as well as to infer a plausible formation mechanism of Pd_1_ SACs. The present results show a straightforward manner to obtain, on a multigram scale, well-defined ligand-free Pd_1_ SACs, which can be effectively used in catalysis under industrially viable reaction conditions without additional reagents. This further demonstrates the great versatility of MOFs and represents a step closer to the real application of MOF-based materials in catalysis.

## Experimental Section

### Preparation of {Cu_6_Sr[(*S*,*S*)-Mecysmox]_3_(OH)_2_(H_2_O)}·15H_2_O (**3**)

(Me_4_N)_2_{Cu_2_[(*S*,*S*)-methox](OH)_2_}·4H_2_O (4.32 g, 6.0 mmol) was dissolved in 50 mL of water. Then, another aqueous solution (10 mL) containing Sr(NO_3_)_2_ (0.42 g, 2.0 mmol) was added dropwise under stirring. After further stirring for 10 h, at room temperature, a green polycrystalline powder was obtained and collected via filtration and dried with ethanol, acetone and diethyl ether. Yield: 2.91 g, 83%. Anal. calcd for C_30_Cu_6_SrH_70_S_6_N_6_O_36_ (1752.2): C, 20.56; H, 4.03; S, 10.98; N, 4.80%. Found: C, 20.51; H, 4.00; S, 10.99; N, 4.83%. IR (KBr): ν = 1605 cm^–1^ (C=O). Well-shaped hexagonal prisms of **1** suitable for X-ray structural analysis could be obtained by slow diffusion in an H-shaped tube of H_2_O/DMF (1:9) solutions containing stoichiometric amounts of (Me_4_N)_2_{Cu_2_[(*S*,*S*)-Mecysmox](OH)_2_}·5H_2_O (0.13 g, 0.18 mmol) in one arm and Sr(NO_3_)_2_ (0.012 g, 0.06 mmol) in the other. They were isolated by filtration on paper and air-dried.

### Preparation of [Pd_2_(H_2_O)(NH_3_)_6_]_0.5_Cl_2_@{Sr^II^Cu^II^_6_[(*S*,*S*)-Mecysmox]_3_(OH)_2_(CH_3_OH)}·12H_2_O (**4**)

Well-formed hexagonal green prisms of **2**, which were suitable for X-ray diffraction, were obtained by soaking crystals of **1** (ca. 25 mg, 0.015 mmol) in a H_2_O/CH_3_OH (1:1) solution of [Pd(NH_3_)_4_]Cl_2_ (0.015 mmol) for 6 h. The process was repeated five times to ensure the maximum loading of [Pd(NH_3_)_4_]Cl_2_. Crystals were washed with a H_2_O/CH_3_OH (1:1) solution several times, isolated by filtration on paper and air-dried. Anal. calcd for C_31_Cl_2_Cu_6_SrH_76_PdS_6_N_9_O_33.5_ (1949.6): C, 19.10; H, 3.93; S, 9.87; N, 6.47%. Found: C, 19.07; H, 3.89; S, 9.91; N, 6.45%. IR (KBr): ν = 1603 cm–1 (C=O).

A multigram-scale procedure was also developed by using the same synthetic procedure but using a higher amount of a polycrystalline sample of **1** (2 g, 1.15 mmol), which were suspended a H_2_O/CH_3_OH (1:1) solution of [Pd(NH_3_)_4_]Cl_2_ (1.1 mmol) for 1 h under a mild stirring. The process was repeated 5 times. Finally, the product was collected by filtration, washed with a H_2_O/CH_3_OH (1:1) solution and air-dried. Anal. calcd for C_31_Cl_2_Cu_6_SrH_76_PdS_6_N_9_O_33.5_ (1949.6): C, 19.10; H, 3.93; S, 9.87; N, 6.47%. Found: C, 19.02; H, 3.87; S, 9.91; N, 6.47%. IR (KBr): ν = 1602 cm^–1^ (C=O).

### (Pd^0^)_0.5_([Pd^II^(H_2_O)(NH_3_)_3_]Cl_2_)_0.5_@{Sr^II^Cu^II^_6_[(*S,S*)-Mecysmox]_3_(OH)_2_(CH_3_OH)}·13H_2_O (**5**)

The same procedure was applied, with the same successful results to both crystals (ca. 25 mg) and a powder polycrystalline sample of **2** (ca. 2 g). They were suspended in H_2_O/CH_3_CH_2_OH (1:1) solutions to which NaBH_4_, divided in 15 fractions (0.4 mmol of NaBH_4_ per mmol of MOF each), were added progressively in the space of 72 h. Each fraction was allowed to react for 1.5 h. After this period, samples were gently washed with a H_2_O/CH_3_OH solution and filtered on paper. Anal. calcd for C_31_ClCu_6_SrH_73.5_PdS_6_N_7.5_O_34.5_ (1906.6): C, 19.53; H, 3.89; S, 10.01; N, 5.51%. Found: C, 19.48; H, 3.87; S, 10.03; N, 5.49%. IR (KBr): ν = 1601 cm^–1^ (C=O).

### Catalysis Details

All reactions were performed under aerobic solvent-free conditions. Palladium acetate (Sigma-Aldrich, >99.8% purity) was weighed (0.13–1.3 mg, which corresponds to 0.03 to 0.30%mol, respectively) in a double-walled 5 mL reactor equipped with a needle connected to a manometer. Then, the corresponding benzyl alcohol (0.2 mL, Sigma-Aldrich, > 98%) was added, and after setting an atmosphere of 4 bar O_2_, the reactor was placed at 150 °C at a stirring rate of 450 rpm for the required reaction time. Aliquots were taken periodically to follow the course of the reaction by GC and by GC-MS, after adding mesitylene (3 μL) as an external standard. Supported Pd nanoparticles (Sigma-Aldrich, 98%) of different loadings 10, 5, and 1% in weight (3.1, 6.1, and 30.7 mg, respectively) and MOF **5** (1% in weight, 30.7 mg) were used as catalysts for the same purpose.

General procedure for the oxidation of 1a with palladium catalysts: In a 10 mL glass vial equipped with a stirring bar, the corresponding benzyl alcohol (1.96 mmol) was charged with the different amounts of the palladium catalyst. The vial was closed with a septum, an oxygen balloon was connected, and the mixture was placed in a preheated metal heating plate at 150 °C and stirred at 450 rpm for the indicated time. Alternatively, we used a lab-made double-walled vial connected to a manometer, where O_2_ can be introduced at the desired pressure through a cannula. After the reaction time, the mixture was analyzed as above.

The following procedure was followed to get the active catalytic species trapped *in situ* after 60 min of reaction, at 150 °C and 4 bar O_2_. Active charcoal was added after depressurization of the reaction, while the reaction mixture was being stirred at reaction temperature. While still hot, the reactor was set aside, and 2 mL of methanol was added to reactor. The mixture was kept in stirring for 10 min. Afterward, the whole mixture was transferred to a 2 mL vial, which was then centrifuged and washed 3 times with 2 mL of fresh methanol each time. After this procedure, the samples were dried at 70 °C under vacuum overnight and then analyzed by HR-TEM. The amount of charcoal used to trap the species (5–30 mg) was calculated in order to obtain a sample with approximately 2–3 wt % of Pd.

### General Procedure for Ultraviolet–Visible (UV–Vis) Spectrophotometric Titrations

In a 10 mL glass vial equipped with a stirring bar, the corresponding benzyl alcohol (1.96 mmol) was charged with 0.006 mmol of palladium acetate. The vial was closed with a septum equipped with a manometer and charged with 4 bar of oxygen. Then, it was placed in a preheated metal heating plate at 150 °C and stirred at 450 rpm for the indicated time. Afterward the mixture was quenched with 4 mL of a 0.02 M triphenylphosphine solution in CH_2_Cl_2_ and analyzed by UV–vis spectrophotometry in quartz cuvettes with an optical path of 10 × 10 mm^2^.

### X-ray Crystallographic Details

Diffraction data for **3** were collected on a Bruker-Nonius X8APEXII CCD area detector diffractometer using graphite-monochromated Mo Kα radiation (λ = 0.71073 Å), whereas data for **4** and **5** were collected using synchrotron radiation at I19 beamline of the Diamond Light Source at λ = 0.6889 Å. Crystal data for **3**–**5**: hexagonal, space group *P*6_3_, *T* = 100(2), *Z* = 2. **3**: C_30_Cu_6_H_70_N_6_O_36_S_6_Sr, *a* = 18.057(4) Å, *c* = 12.800(3) Å, *V* = 3614.6(17) Å^3^; **4**: C_31_Cu_6_H_76_N_9_O_33.5_S_6_SrPdCl_2_, *a* = 17.86780(10) Å, *c* = 12.80840(10) Å, *V* = 3541.34(5) Å^3^; **5**: C_31_Cu_6_H_73.5_N_7.5_O_34.5_S_6_SrPdCl, *a* = 17.8206(2) Å, *c* = 12.7821(3) Å, *V* = 3515.42(11) Å^3^. Further details can be found in the Supporting Information. CCDC 1995182, 1995183, and 1995184 for **3**, **4**, and **5**, respectively, contain the supplementary crystallographic data for this paper. These data can be obtained free of charge via www.ccdc.cam.ac.uk/conts/retrieving.html or from the Cambridge Crystallographic Data Centre, 12 Union Road, Cambridge CB21EZ, UK; fax: (+44)1223–336–033; or deposit@ccdc.cam.ac.uk.

### X-ray Powder Diffraction Measurements

Polycrystalline samples of **3**, **4**, and **5** were introduced into 0.5 mm borosilicate capillaries prior to being mounted and aligned on a Empyrean PANalytical powder diffractometer, using Cu Kα radiation (λ = 1.54056 Å). For each sample, five repeated measurements were collected at room temperature (2θ = 2–60°) and merged in a single diffractogram. A polycrystalline sample of **5** was also measured after catalysis following the same procedure.

### X-ray Photoelectron Spectroscopy (XPS) Measurements

Samples **4**, **5**, and **5′** (after catalysis) were prepared by sticking, without sieving, the MOF onto a molybdenum plate with cellophane tape, followed by air drying. Measurements were performed on a K-Alpha X-ray Photoelectron Spectrometer (XPS) System using a monochromatic Al K(alpha) source (1486.6 eV). As an internal reference for the peak positions in the XPS spectra, the C 1s peak has been set at 284.8 eV.

### FTIR Spectroscopy of Adsorbed CO

Fourier transform infrared (FTIR) using CO as a probe molecule was used to evaluate electronic properties of MOF **5**. Spectra were recorded once complete coverage of CO at the specified CO partial pressure was achieved. See the Supporting Information for details.

### Computational Methods

Periodic DFT calculations were performed with the Vienna Ab-initio Simulation Package (VASP) code, using the Perdew–Burke–Ernzerhof (PBE) exchange-correlation functional. See Supporting Information for details.

### XAS Techniques

XANES and EXAFS measurements of Pd foil, Pd acetate, and MOF **5** were carried out on CLAESS beamline at ALBA Synchrotron Light Source, Barcelona, Spain. See the Supporting Information for details.

## Supporting Information Available

Physical techniques. Crystallographic and catalytic details. Tables S1–S8. Figures S1–S31. (PDF)
