# Supplementary material for: Soluble/MOF-Supported Palladium Single Atoms Catalyze the Ligand-, Additive-, and Solvent-Free Aerobic Oxidation of Benzyl Alcohols to Benzoic Acids
Source: J Am Chem Soc. 2021 Feb 4;143(6):2581–92. doi: 10.1021/jacs.0c12367 (PMC9645712; doi:10.1021/jacs.0c12367)
Supplement: Supplementary file 1 — ja0c12367_si_001.pdf [file ja0c12367_si_001.pdf]

Supporting Information (SI) for the manuscript:

Soluble/MOF-supported Palladium Single Atoms Catalyze the Ligand-, Additive- and Solvent-free Aerobic Oxidation of Benzyl Alcohols to Benzoic Acids

Estefanía Tiburcio,[†,◊] Rossella Greco, [‡,◊] Marta Mon,[‡,◊] Jordi Ballesteros-Soberanas,[‡] Jesús Ferrando–Soria,[†] Miguel López-Haro,[§,♣] Juan Carlos Hernández-Garrido,[§,♣] Judit Oliver-Meseguer,[‡] Carlo Marini,[††] Mercedes Boronat,[‡] Donatella Armentano,[*,♠] Antonio Leyva–Pérez[*, ‡] and Emilio Pardo[*,†]

[†]Instituto de Ciencia Molecular (ICMol), Universidad de Valencia, 46980 Paterna, Valencia, Spain.

[‡]Instituto de Tecnología Química (UPV–CSIC), Universitat Politècnica de València–Consejo Superior de Investigaciones Científicas, Avda. de los Naranjos s/n, 46022 Valencia, Spain.

[§]Departamento de Ciencia de los Materiales e Ingeniería Metalúrgica y Química Inorgánica, Facultad de Ciencias, Universidad de Cádiz, Campus Universitario de Puerto Real, 11510 Puerto Real, Cádiz, Spain.

[♣]Instituto Universitario de Investigación en Microscopía Electrónica y Materiales (IMEYMAT). Facultad de Ciencias. Universidad de Cádiz, Campus Universitario de Puerto Real, 11510 Puerto Real, Cádiz, Spain.

[††]CELLS–ALBA Synchrotron, E-08290 Cerdanyola del Vallès, Barcelona, Spain.

[♠]Dipartimento di Chimica e Tecnologie Chimiche (CTC), Università della Calabria, Rende 87036, Cosenza, Italy.

Experimental Section

Materials. All chemicals were of reagent grade quality. They were purchased from commercial sources and used as received.

H_2Me_2-(*S*,*S*)-Mecysmox = bis[(*S*)-methyl-(*L*)-cysteine]oxalyl diamide. The proligand was prepared using the following synthetic procedure: First, under a N_2 atmosphere, an excess of thionyl chloride (13.10 mL, 180 mmol) was added dropwise, under stirring at 0 °C on an ice-bath, to a solution of (*S*)-methyl-(*L*)-cysteine amino acid (8.11 g, 60 mmol) in 150 mL of MeOH. The resulting colorless solution was refluxed for 6 hours. Then, the excess of thionyl chloride was distilled with MeOH (3 x 150 mL). The reaction mixture was washed with acetone (150 mL) and diethyl ether (100 mL) and further concentrated, under reduced pressure, to afford the methyl ester derivative of the (*S*)-methyl-(*L*)-cysteine amino acid, which was used in the next step without further purification.

Second, the resulting methyl ester derivative of the (*S*)-methyl-(*L*)-cysteine amino acid (8.95 g, 60 mmol) was dissolved in 250 mL of dichloromethane and charged with triethylamine (8.4 mL, 60 mmol). To the resulting colorless reaction mixture, was added dropwise another solution containing oxalyl chloride (2.54 mL, 30.0 mmol) in dichloromethane (150 mL) under vigorous stirring at 0 °C on an ice-bath. The resulting solution was further stirred during two hours. The small amount of white solid (Et_3NHCl) formed was filtered off and the resulting solution was then concentrated in a rotatory evaporator to a final volume of 100 mL. The pale yellow solution was washed three times with water (3x50 mL) and finally, the solvent was removed in a rotatory evaporator to afford a white solid, which was collected with water and dried under vacuum. Yield: 9.62 g, 91%; Anal. calcd (%) for $C_{12}H_{20}S_2N_2O_6$ (352.4): C 40.98, H 5.72, S 18.20, N 7.95; found: C 40.97, H 5.68, S 18.26, N 7.99; ^{1}H NMR ([D_6]DMSO):

2.20 (s, 6H; SCH_3), 2.97 (m, 2H; CH_2), 3.17 (m, 2H; CH_2), 3.62 (s, 6H; OCH_3), 4.78 (t, 2H; CH), 9.01 (d, 2H; NH from CONH). IR (KBr): ν = 1763, 1751and 1656 cm^{-1} (C=O).

$(Me_4N)_2\{Cu_2[(\textit{S,S})\text{-Mecysmox}](OH)_2\} \cdot 5H_2O$: An aqueous suspension (60 mL) of H_2Me_2-(S,S)-Mecysmox (10.572 g, 30 mmol) was treated with a 25% methanolic solution of Me_4NOH (36 mL, 125 mmol) until complete dissolution. Another aqueous solution (25 mL) of $CuCl_2$ (8.07 g, 60 mmol) was then added dropwise while the reaction mixture was stirred. The resulting deep green solution was concentrated to a volume of *ca.* 5-10 mL in a rotary evaporator affording a green polycrystalline solid that was gently washed with acetone filtered off and dried under vacuum. Yield: 14.77 g, 68%; Anal.: calcd for $C_{18}H_{48}Cu_2S_2N_4O_{13}$ (719.8): C, 30.03; H, 6.72; S, 8.91; N, 7.78%. Found: C, 30.13; H, 6.63; S, 8.93; N, 7.75%. IR (KBr): ν = 3621 cm^{-1} (O-H), 3023, 2964 cm^{-1} (C-H), 1608 cm^{-1} (C=O).

$\{Cu_6Sr[(\textit{S,S})\text{-Mecysmox}]_3(OH)_2(H_2O)\} \cdot 15H_2O$ (3): $(Me_4N)_2\{Cu_2[(S,S)\text{-methox}](OH)_2\} \cdot 4H_2O$ (4.32 g, 6.0 mmol) was dissolved in 50 mL of water. Then, another aqueous solution (10 mL) containing $Sr(NO_3)_2$ (0.42 g, 2.0 mmol) was added dropwise under stirring. After further stirring for 10 h, at room temperature, a green polycrystalline powder was obtained and collected *via* filtration and dried with ethanol, acetone and diethyl ether. Yield: 2.91 g, 83%; Anal.: calcd for $C_{30}Cu_6SrH_{70}S_6N_6O_{36}$ (1752.2): C, 20.56; H, 4.03; S, 10.98; N, 4.80%. Found: C, 20.51; H, 4.00; S, 10.99; N, 4.83%. IR (KBr): ν = 1605 cm^{-1} (C=O). Well-shaped hexagonal prisms of **3** suitable for X-ray structural analysis could be obtained by slow diffusion, in an H-shaped tube, of H_2O/DMF (1:9) solutions containing stoichiometric amounts of $(Me_4N)_2\{Cu_2[(S,S)\text{-Mecysmox}](OH)_2\} \cdot 5H_2O$ (0.13 g, 0.18 mmol) in one arm and $Sr(NO_3)_2$ (0.012 g, 0.06 mmol) in the other. They were isolated by filtration on paper and air-dried.

$[Pd_2(H_2O)(NH_3)_6]_{0.5}Cl_2$@$\{Sr^{II}Cu^{II}_6$[(*S*,*S*)–Mecysmox$]_3(OH)_2(CH_3OH)\}$ · $12H_2O$ (4): Well–formed hexagonal green prisms of **4**, which were suitable for X–ray diffraction, were obtained by soaking crystals of **3** (*ca.* 25 mg, 0.015 mmol) in a H_2O/CH_3OH (1:1) solution of $[Pd(NH_3)_4]Cl_2$ (0.015 mmol) for 6 hours. The process was repeated five times to ensure the maximum loading of $[Pd(NH_3)_4]Cl_2$. Crystals were washed with a H_2O/CH_3OH (1:1) solution several times, isolated by filtration on paper and air–dried. Anal.: calcd for $C_{31}Cl_2Cu_6SrH_{76}PdS_6N_9O_{33.5}$ (1949.6): C, 19.10; H, 3.93; S, 9.87; N, 6.47%. Found: C, 19.07; H, 3.89; S, 9.91; N, 6.45%. IR (KBr): ν = 1603 cm–1 (C=O).

A multigram scale procedure was also developed by using the same synthetic procedure but using a higher amount of a polycrystalline sample of **3** (2 g, 1.15 mmol), which were suspended a H_2O/CH_3OH (1:1) solution of $[Pd(NH_3)_4]Cl_2$ (1.1 mmol) for 1 hour under a mild stirring. The process was repeated 5 times. Finally, the product was collected by filtration, washed with a H_2O/CH_3OH (1:1) solution and air–dried. Anal.: calcd for $C_{31}Cl_2Cu_6SrH_{76}PdS_6N_9O_{33.5}$ (1949.6): C, 19.10; H, 3.93; S, 9.87; N, 6.47%. Found: C, 19.02; H, 3.87; S, 9.91; N, 6.47%. IR (KBr): ν = 1602 cm-1 (C=O).

$(Pd^0)_{0.5}([Pd^{II}(H_2O)(NH_3)_3]Cl_2)_{0.5}$@$\{Sr^{II}Cu^{II}_6$[(*S*,*S*)-Mecysmox$]_3(OH)_2(CH_3OH)\}$ · $13H_2O$ (5): The same procedure was applied, with the same successful results to both, crystals (*ca.* 25 mg) and a powder polycrystalline sample of **4** (*ca.* 2 g). They were suspended in H_2O/CH_3CH_2OH (1:1) solutions to which $NaBH_4$, divided in 15 fractions (0.4 mmol of $NaBH_4$ per mmol of MOF each), were added progressively in the space of 72 hours. Each fraction was allowed to react for 1.5 hour. After this period, samples were gently washed with a H_2O/CH_3OH solution and filtered on paper. Anal.: calcd for $C_{31}ClCu_6SrH_{73.5}PdS_6N_{7.5}O_{34.5}$ (1906.6):

C, 19.53; H, 3.89; S, 10.01; N, 5.51%. Found: C, 19.48; H, 3.87; S, 10.03; N, 5.49%. IR (KBr): ν = 1601 cm^{-1} (C=O).

Physical Techniques. Elemental (C, H, S, N) analyses were performed at the Microanalytical Service of the Universitat de València. ^1H NMR spectra were recorded at room temperature on a Bruker AC 200 (200.1 MHz) spectrometer. FT–IR spectra were recorded on a Perkin-Elmer 882 spectrophotometer as KBr pellets. The thermogravimetric analyses were performed on crystalline samples under a dry N_2 atmosphere with a Mettler Toledo TGA/STDA 851e thermobalance operating at a heating rate of 10 ºC min^{-1}. ICP-MS analyses were carried out with an inductively coupled plasma atomic emission spectrometer with an Agilent 7900 mass detector (Microanalytical Service of the Universitat de València).

Gas adsorption. The N_2 adsorption–desorption isotherms at 77 K were carried out on crystalline samples of **3**, **4** and **5** with a Micromeritics ASAP2020 instrument. Samples were evacuated at 70 ºC during 24 hours under 10-6 Torr prior to their analysis.

X–ray Powder Diffraction Measurements. Polycrystalline samples of **3**, **4** and **5** were introduced into 0.5 mm borosilicate capillaries prior to being mounted and aligned on a Empyrean PANalytical powder diffractometer, using Cu Kα radiation (λ = 1.54056 Å). For each sample, five repeated measurements were collected at room temperature (2θ = 2–60°) and merged in a single diffractogram. A polycrystalline sample of **5** was also measured after catalysis following the same procedure.

X–ray photoelectron spectroscopy (XPS) measurements. Samples **4** and **5** were prepared by sticking, without sieving, the MOF onto a molybdenum plate with scotch tape film, followed by air drying. Measurements were performed on a K-Alpha™ X-ray Photoelectron Spectrometer (XPS) System using a monochromatic Al

K(alpha) source (1486.6 eV). As an internal reference for the peak positions in the XPS spectra, the C1s peak has been set at 284.8 eV.

Microscopy measurements. Electron microscopy studies were performed on a FEI Titan Themis 60–300 Double Aberration Corrected microscope working at 200kV. The aberrations of the condenser lenses were corrected up to fourth–order using the Zemlin tableau to obtain a sub–Angstrom electron probe. A condenser aperture of 50 µm yielding an electron probe with a convergence angle of 20 mrad was used. In order to avoid sample modification under the electron probe a beam current of 0.025 nA was used. Chemical XEDS maps were collected at medium magnifications (c.a. 100k to 200k) by STEM-XEDS using the high-efficiency SuperX G2 detection system equipped in the microscope, which integrates four windowless detectors surrounding the sample and high performance signal-procedure hardware. STEM-XEDS was used to map the spatial distribution Pd species. To limit the damage by the electron beam, a fast image recording protocol was used by combining a beam current of 25 pA, and 4µs dwell time and an automated fine-tuning alignment of A1 and C1 using the OptiSTEM software.

X-ray crystallographic data collection and structure refinement. Crystals of **3**, **4** and **5** with 0.14 x 0.10 x 0.10, 0.06 x 0.05 x 0.05, and 0.05 x 0.05 x 0.05 as dimensions were selected and mounted on a MiTeGen MicroMount in Paratone oil and very quickly placed on a liquid nitrogen stream cooled at 100 K, to avoid the possible degradation upon dehydration or exposure to air. Diffraction data for **3** were collected on a Bruker-Nonius X8APEXII CCD area detector diffractometer using graphite-monochromated Mo-K_{α} radiation (λ = 0.71073 Å), whereas data for **4** and **5** using synchrotron radiation at I19 beamline of the Diamond Light Source at λ = 0.6889 Å. The data were processed through SAINT[1] reduction and SADABS[2] multi-scan absorption (**3**) or xia2[3] (**4** and **5**) software. The structures were solved with the SHELXS structure solution program,

using the Patterson method. The model was refined with version 2018/3 of SHELXL against F^2 on all data by full-matrix least squares.[3]

As reported in the main text, the robustness of the 3D network, allowed the resolution of the crystal structure of both **4** and **5** adsorbates, being their crystals suitable for X–ray diffraction, even over one– and two–step process, after a crystal–to–crystal transformation. For these reasons it is reasonable to observe a diffraction pattern sometimes affected by expected internal imperfections of the crystals [likely at the origin of some Alert level B for **4-5** in checkcifs related to U(eq) value of some atoms] and thus a quite expected difficulty to perform a perfect correction of anisotropy, mainly affected by highly flexible thioether chains as terminal moiety (vide infra).

In all samples, all non–hydrogen atoms of the MOF net, except the dimethyl thioether chains from the mecysmox ligand, and Pd atoms (**4** and **5**), were refined anisotropically. The use of some C-C and C-S bond lengths restrains as well as Pd-S (**5**), during the refinements, has been reasonable imposed and related to extraordinary flexibility of dimethyl thioether chains from the methylcysteine residues, that are dynamic components of the frameworks (see Figures S8 and S9). In the refinement of crystal structures some further restrains, to make the refinement more efficient, have been applied, for instance ADP components have been restrained to be similar to other related atoms, using SIMU 0.04 for disordered sections or EADP for group of atoms expected to have essentially similar ADPs. All the hydrogen atoms of the net were set in calculated position and refined isotropically using the riding model. Disordered sites for atoms C4S in refinement of **4** and **5**, belonging to the dimethyl thioether chains, reside in special position resulting statistically disordered for symmetry reason.

The occupancy factors of Pd atoms have been defined in agreement with SEM and ICP–MS results (see Table S2). The high thermal factors are most likely related to the porosity of the net hosting Pd^{2+} ions/Pd^0 atoms within very large pores.

The solvent molecules in **3-5** were highly disordered and have not been found from the ΔF map neither the NH_3 molecules and Cl^- anions in **4-5** belonging to the mononuclear complex $[Pt(NH_3)_4]Cl_2$ inserted within pores. The quite large channels featured by this series of MOFs likely account for that [see Figures S9 and S11]. Consequently, in **3-5**, the contribution to the diffraction pattern from the highly disordered water and NH_3 molecules/Cl^- anions located in the voids was subtracted from the observed data through the SQUEEZE method, implemented in PLATON.[5]

A summary of the crystallographic data and structure refinement for the three compounds is given in Table S3. The comments for the alerts A and B are described in the CIFs using the validation reply form (vrf). CCDC reference numbers are 1995182-1995184 for **3-5**, respectively.

The final geometrical calculations on free voids and the graphical manipulations were carried out with PLATON[5] implemented in WinGX,[6] and CRYSTAL MAKER[7] programs, respectively.

FTIR spectroscopy of adsorbed CO. Fourier transform infrared (FTIR) using CO as a probe molecule was used to evaluate electronic properties of MOF **5**. The experiments have been carried out in a homemade IR cell able to work in the high and low (77 K) temperature range. Prior to CO adsorption experiments, the sample was evacuated at 298 K under vacuum (10^{-6} mbar) for 1 h. CO adsorption experiments were performed at 77 K in the 0.2–20 mbar range. Spectra were recorded once complete coverage of CO at the specified CO partial pressure was achieved. Deconvolution of the

IR spectra has been performed in the Origin software using Gaussian curves where the full width at half–maximum (fwhm) of the individual bands has been taken as constant. The peak areas are normalized to the sample weight.

Computational details. Periodic density functional theory (DFT calculations were performed with the Vienna Ab-initio Simulation Package (VASP) code,[8] using the Perdew-Burke-Ernzerhof (PBE) exchange-correlation functional.[9] The valence density was expanded in a plane wave basis set with a kinetic energy cutoff of 600 eV, and the effect of the core electrons in the valence density was taken into account by means of the projected augmented wave (PAW) formalism.[10] Integration in the reciprocal space was carried out at the Γ k-point of the Brillouin zone. During geometry optimizations, the positions of all atoms in the system were fully relaxed without any restriction by means of a conjugate-gradient algorithm until atomic forces were smaller than 0.02 eV/Å, and harmonic vibrational frequencies were calculated numerically. Atomic charges were estimated using the theory of atoms in molecules (AIM) of Bader.[11]

Interaction energies between CO and Pd in different environments were calculated as

E_{int} = E(CO-Pd) – E(CO) – E(Pd)

Where E(CO-Pd) is the total energy of the system after CO adsorption, E(CO) is the total energy of an isolated CO molecule in a 20x20x20 $Å^3$ cubic box, and E(Pd) is the total energy of the corresponding Pd model without CO adsorbed.

XAS techniques. XANES and EXAFS measurements were carried out on CLAESS beamline at ALBA Synchrotron Light Source, Barcelona, Spain.[12] The synchrotron light coming from the multipole wiggler has been first vertically collimated, then monochromatized using two pairs of liquid nitrogen cooled Si(311) crystals and finally focused on the sample position down to ~500×500 μm^2. Rh stripe coating on the two

optical mirrors guarantees the higher harmonics rejection. A 4 μm-thick Pd foil (Goodfellow, 99.99%) has been used for calibrating the energy scale, checking the alignment of scans, and determining the passive electron reduction factor in following data post-processing. Samples preparation consisted in 13 mm pellets with cellulose for Pd foil, Pd acetate and MOF **5**. Pd in solution sample was prepared in a 10 ml glass vial equipped with a stirring bar, the corresponding benzyl alcohol (1.96 mmol, 200 µl) was charged with 0.08% mol of $Pd(OAc)_2$ (0.4 mg). The vial was closed with a septum equipped with a manometer and charged with air. Then the mixture was placed in a pre-heated metal heating plate at 150 °C and stirred at 450 rpm for 30 min. The XAS data were obtained in fluorescence mode at the Pd K-edge (24.350 keV) for our samples and transmission mode for the standards, placing the samples at 45 deg geometry (with respect the incoming beam) by means of a 6 elements Silicon Drift detector from Quantum Detector. No radiation damage was observed during the measurements. Energy scale has been calibrated by measuring a Pd foil. Several scans were acquired in continuous mode to ensure spectral reproducibility and good signal-to-noise ratio. Data reduction has been done using the Demeter program suite:[13] raw data has been normalized by subtracting and dividing pre-edge and post-edge backgrounds as low order polynomial smooth curves. The local structure of the sample has been than refined using the EXAFS signal in the k range 3:10 $Å^{-1}$. Corresponding Fourier transformed curved are shown in Figure 2. Theoretical models for further data analysis have been obtained from palladium NPs and PdS structures using the FEFF6 code and fitted[14] to the EXAFS spectra by adjusting the structural parameters (i.e. coordination number, distances, disorder factors).

Catalysis

Typical procedure for the oxidation of benzyl alcohol 1a in open air. In a 10 ml glass vial equipped with a stirring bar, benzyl alcohol 1a (1.96 mmol) was charged with the palladium catalyst, and the vial was connected to a condenser. The mixture was placed in a pre-heated metal heating plate at 150°C and stirred at 450 rpm for the indicated time. Aliquots were extracted from the reaction at different times, mesitylene (3 ml) was added as an external standard, and the mixture was analysed by GC.

Table S1. Catalytic results for different Pd compounds.

Catalyst	2a	Benzaldehyde	Other products
$Pd(OAc)_2$	75%	3.6%	6.9%
K_2PdCl_4	55.6%	15.5%	15.7%
$Pd_2(dba)_3$*	37.9%	18.2%	9.9%
$Pd(acac)_2$	51.4%	15.8%	9.6%
Pd_1 SACs (MOF **5**)	41.1%	26.4%	7.6%
$Pd(PPh_3)_4$	7.2%	24.1%	8.8%
$Pd(PPh_3)_2Cl_2$	0.7%	4.5%	7.0%

Reaction conditions: 1.96 mmol substrate, 0.3% mol Pd, 4 atm O2, 150°C, 450 rpm, 4 h. GC yields. * 15 h

Table S2 EXAFS results for Pd(0) foil, Pd SACs in solution and supported in the MOF.

Sample	NPd-Pd	NPd-S	σ^2 (Å^2)	ΔE0 (eV)	R
**Pd foil**	12 (fixed)	--	0.005 ±0.0004	3.03 ± 1.01	2.736 ± 0.007
**Pd in solution**	6.25 ± 1.62	--	0.006 ±0.002	3.56 ±1.80	2.759 ± 0.013
**Pd SAC-MOF**	--	3.77 ± 0.45	0.003 ±0.0008	-2.95 ±1.47	2.291 ± 0.014

Table S3. Selected data from the ICP–MS[a] and SEM/EDX[b] analyses.

Compound 2

**Metal**	*% mass*[a]	*Metal stoichiometry*[a]	*% mass*[b]	*Metal stoichiometry*[b]
**Cu**	16.71	6.00	16.99	6.00
**Sr**	1.77	1.01	1.58	0.88
**Pd**	17.31	2.03	17.98	2.07

Compound 3

**Metal**	*% mass*[a]	*Metal stoichiometry*[a]	*% mass*[b]	*Metal stoichiometry*[b]
**Cu**	16.94	6.00	17.12	6.00
**Sr**	1.78	0.99	1.43	0.79
**Pd**	17.34	2.00	18.34	2.10

[a]Solid samples were digested with 0.5 mL of HNO_3 69% at 60°C for 4 hours followed by the addition of 0.5 mL of HCl 37% and digestion 80°C for 1 hour. The metal stoichiometry was calculated in both cases with respect to Cu values.

Table S4. Summary of Crystallographic Data for {Cu_6Sr[(*S*,*S*)-Mecysmox]$_3(OH)_2(H_2O)$} · $15H_2O$ **(3),** $[Pd_2(H_2O)(NH_3)_6]_{0.5}Cl_2$@{$Sr^{II}Cu^{II}_6$[(*S*,*S*)–Mecysmox]$_3(OH)_2(CH_3OH)$} · $12H_2O$ **(4)** and $(Pd^0)_{0.5}([Pd^{II}(H_2O)(NH_3)_3]Cl_2)_{0.5}$@{$Sr^{II}Cu^{II}_6$[(*S*,*S*)-Mecysmox]$_3(OH)_2(CH_3OH)$} · $13H_2O$ **(5).**

Compound	**3**	**4**	**5**
Formula	$C_{30}Cu_6H_{70}N_6O_{36}S_6$ Sr	$C_{31}Cu_6H_{76}N_9O_{33.5}S_6SrPdCl_2$	$C_{31}Cu_6H_{73.5}N_{7.5}O_{34.5}S_6SrPdCl$
M (g mol^{-1})	1752.14	1949.52	1906.54
λ (Å)	0.71073	0.6889	0.6889
Crystal system	Hexagonal	Hexagonal	Hexagonal
Space group	$P6_3$	$P6_3$	$P6_3$
a (Å)	18.057(4)	17.86780(10)	17.8206(2)
c (Å)	12.800(3)	12.80840(10)	12.7821(3)
V (Å^3)	3614.6(17)	3541.34(5)	3515.42(11)
Z	2	2	2
ρ_{calc} (g cm^{-3})	1.610	1.828	1.801
μ (mm^{-1})	2.719	2.760	2.742
T (K)	100	100	100
θ range for data collection (°)	1.302 to 26.192	0.971 to 36.024	2.005 to 35.956
Completeness to θ = 25.0	100%	100%	100
Measured reflections	44294	77740	76776
Unique reflections (Rint)	4808 (0.1620)	11688 (0.0424)	11614 (0.1080)
Observed reflections [$I > 2\sigma(I)$]	2375	8552	3420
Goof	0.994	1.293	0.916
R^a [$I > 2\sigma(I)$] (all data)	0.0752(0.1446)	0.0566 (0.0702)	0.0694 (0.1616)
wR^b [$I > 2\sigma(I)$] (all data)	0.1809(0.2007)	0.1783(0.1858)	0.1819 (0.1983)
CCDC	1995182	1995183	1995184

^{a} $R = \sum(|F_o| - |F_c|)/\sum|F_o|$. ^{b} $wR = [\sum w(|F_o| - |F_c|)^2/\sum w|F_o|^2]^{1/2}$.

Table S5. DFT data for Pd^0 atoms and $PdCl_2$ within the MOF.

Site	Ligands	Location	qPd (e)	rPd-S (Å)	rPd-O (Å)
**Pd0-A**	O, S	channel	0.0405	2.206	2.144
**Pd0-B**	O, S	interstitial	0.1408	2.192	2.190
**Pd0-C**	S, S	channel	-0.0467	2.270	2.263[a]
**$PdCl_2$**	O, S	channel	0.6658	2.246	2.242

[a] rPd-S

Table S6. DFT data for the interaction of CO with Pd(0) atoms and $PdCl_2$ within the MOF, and with isolated Pd_{13} and $PdCl_2$ (gas) for comparison. Pd0-A-C refers to the different interaction models presented in Figure 7 in the main text.

	**E_{int} CO (kcal/mol)**	**ν(CO)[a] (cm^{-1})**	**rPd-C (Å)**	**rCO (Å)**	**qPd (e)**	**rPd-S (Å)**	**rPd-O (Å)**
**Pd0-A**	-39.0	1997	1.796	1.167	0.1649	2.393	4.680
**Pd0-B**	-28.9	1931	1.846	1.173	0.3435	2.494	2.265
**Pd0-C**	-31.1	1944	1.835	1.172	0.2303	2.429	2.406
**$PdCl_2$**	-0.5	2083	2.864	1.148	0.6778	2.258	2.242
**Pd_{13}**	-43.63	1999	1.836	1.163	0.1642		
**$PdCl_2$ (gas)**	-42.15	2125	1.801	1.153	0.817		

[a] The calculated frequency For CO (gas) is 2141 cm^{-1}, in good agreement with the experimental value of 2143 cm^{-1}.

Table S7. Effect of Cu into the catalytic reaction.

Entry	Catalyst	2	Benzaldehyde	Other products
1	$Pd(OAc)_2$	75%	3.6%	6.9%
2	$Cu(OAc)_2$	0.6%	2.8%	0.2%
3	$Pd(OAc)_2/Cu(OAc)_2$	38.7%	33.4%	11.4%
4	Pd^0_1 SACs-MOF	41.1%	26.4%	7.6%
5	MOFCu	0.8%	5.0%	9.9%

Table S8. Comparison of the turnover frequency (TOF) for previously reported catalysts and the catalyst reported here for the aerobic oxidation of benzyl alcohol **1a** and cinnamyl alcohol to the corresponding acids.

Entry	Product	Catalyst	Conditions	Additives/Solvent	TOF (min^{-1})	Reference
1		Pd(II)-biquinoline	air (40 atm) 125°C	$NaOAc{\cdot}3H_2O$, NaOH/ H_2O	$8{\cdot}10^{-2}$	18
2		Azobenzene	O_2 (1 atm) 80°C	NaOH/1,4-dioxane	$2{\cdot}10^{-4}$	22
3	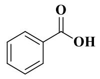	Au@PVP	air (1 atm) 25°C	KOH/H_2O	0.12	24
4		Pd@P-E15	O_2 (1 atm) 90°C	K_2CO_3/ H_2O	0.21	28
5		$Pd(OAc)_2$	O_2 (4 atm) 150°C	-	7.95	This work
6	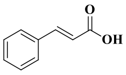	Au@CeO_2	O_2 (1 atm) 80°C	Na_2CO_3/ H_2O	0.82	23

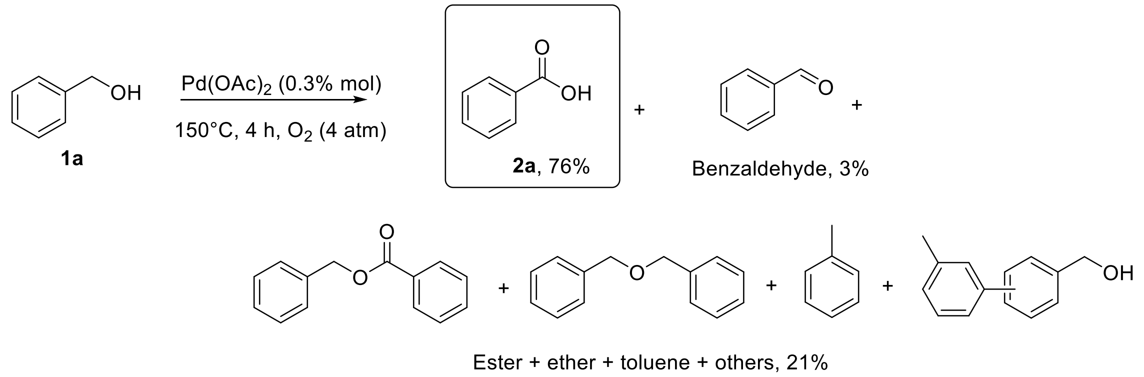

Figure S1. Yields for the aerobic oxidation of neat benzyl alcohol **1a** to benzoic acid **2a** catalyzed by $Pd(OAc)_2$ at 150 ºC and 4 bars of O_2.

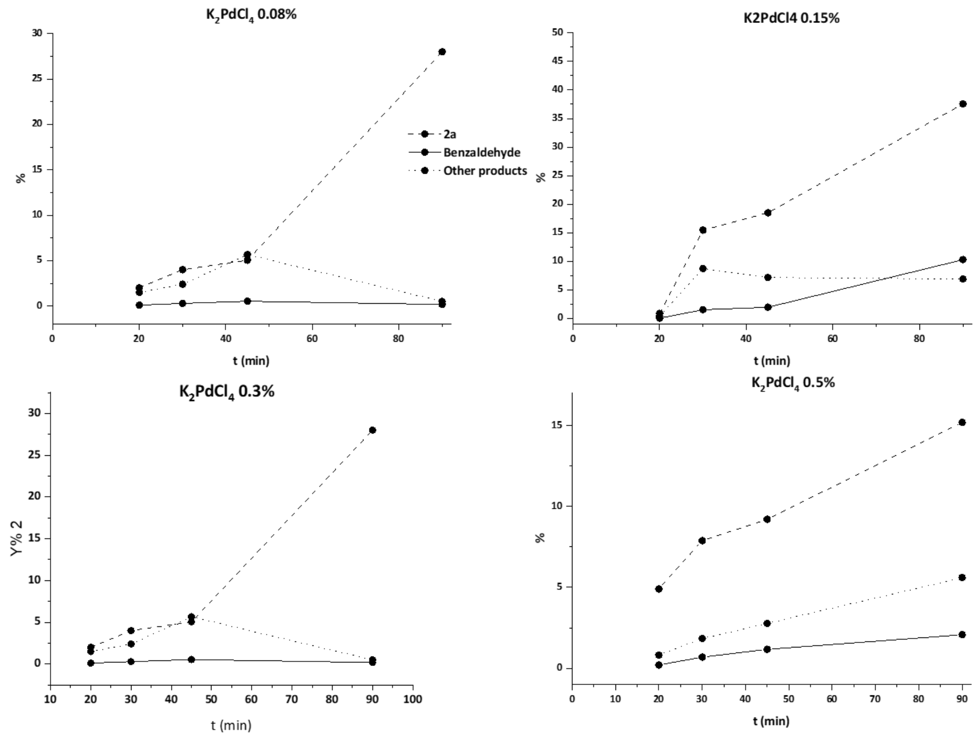

Figure S2. Catalytically active Pd sources for the aerobic oxidation of neat benzyl alcohol **1a** to benzoic acid **2a** at 150 ºC and 4 bars of O_2.

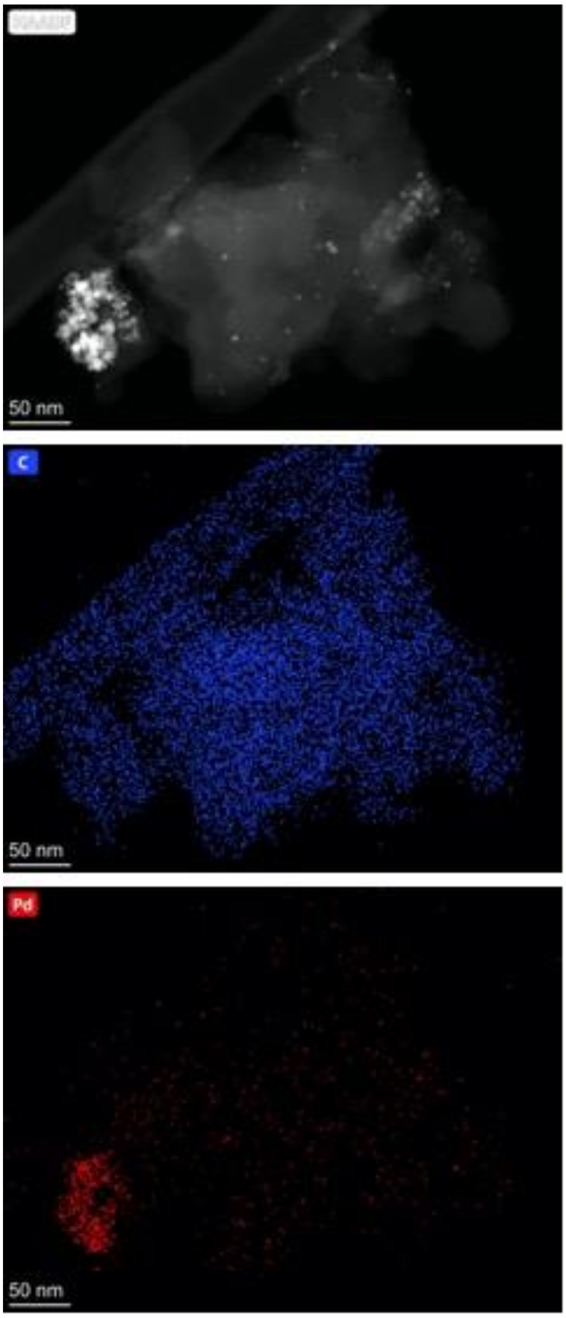

Figure S3. Elemental STEM-XEDS mapping of the Pd species in solution during reaction, after being trapped in active charcoal.

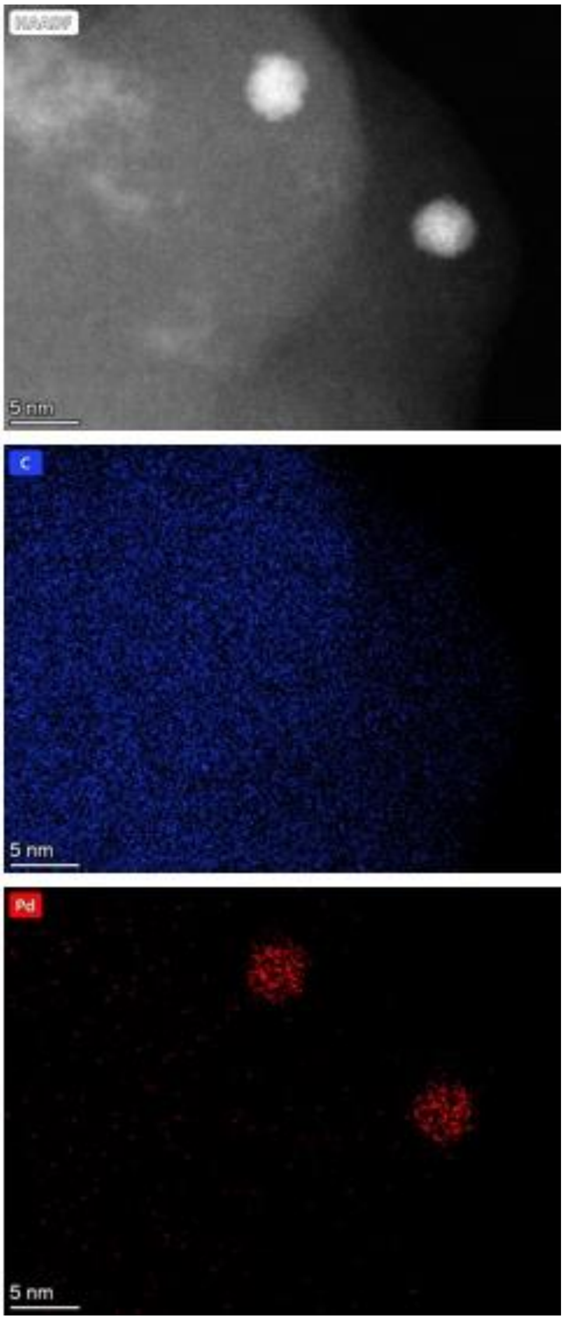

Figure S4. Higher magnification for the elemental STEM-XEDS mapping of the Pd species in solution during reaction, after being trapped in active charcoal.

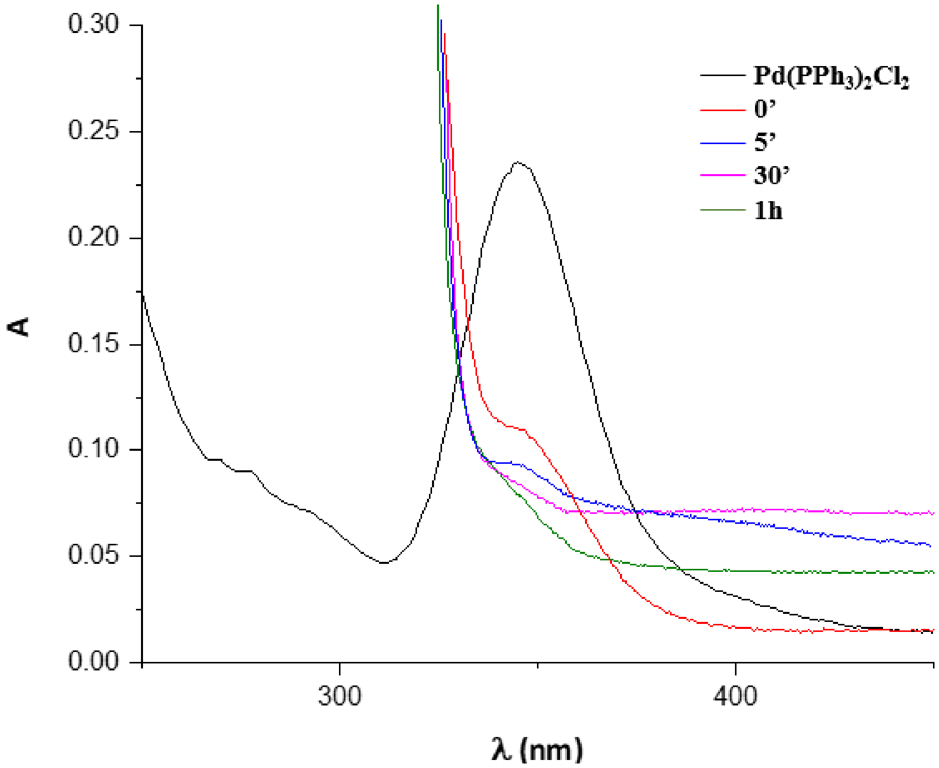

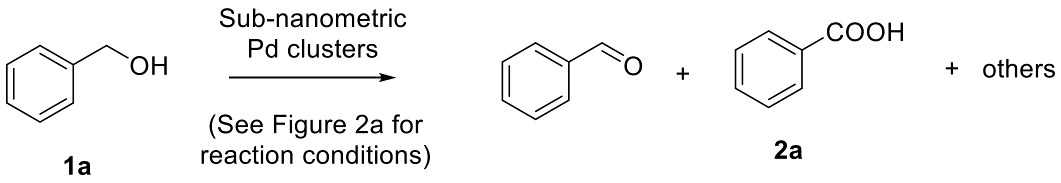

Pd_{3-4} in DMF	40.5 %	2.7 %	2.2 %
Pd_{3-5} in EVOH	5.1 %	0.0 %	2.1 %

Figure S5. Top: Ultraviolet-visible (UV-Vis) spectrophotometric titrations with PPh_3 during the oxidation of neat benzyl alcohol **1a** to benzoic acid **2a** at 150 ºC and 4 bars of O_2, catalyzed by $Pd(OAc)_2$. Complex $Pd(PPh_3)_2Cl_2$ is used as a reference. Complex $Pd(PPh_3)_4$ absorbs at higher energies. Bottom: Yields for the aerobic oxidation of neat benzyl alcohol **1a** to benzoic acid **2a** catalyzed by two differently prepared Pd clusters in solution (0.1 mol%), at 150 ºC and 4 bars of O_2.

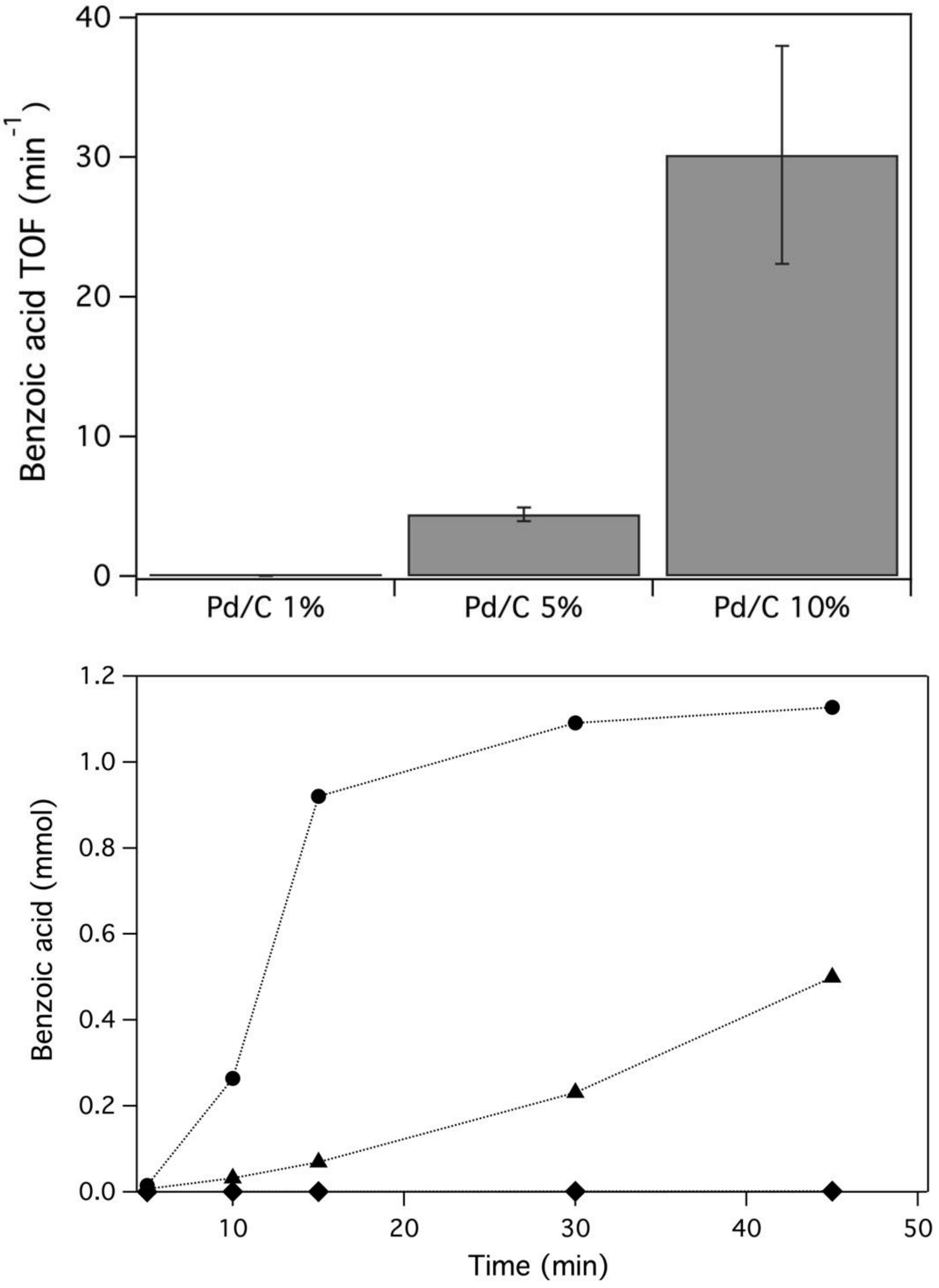

Figure S6. Oxidation of neat benzyl alcohol **1a** to benzoic acid **2a** at 150 ºC and 4 bars of O_2, catalyzed by commercially available samples of Pd/C (1-10 wt% Pd loading and 5-50 nm average particle diameter). The leached amount of Pd in solution is the same for all three initial rate experiments. TOF (top) and representative kinetic profile for the formation of benzoic acid **2a** (bottom).

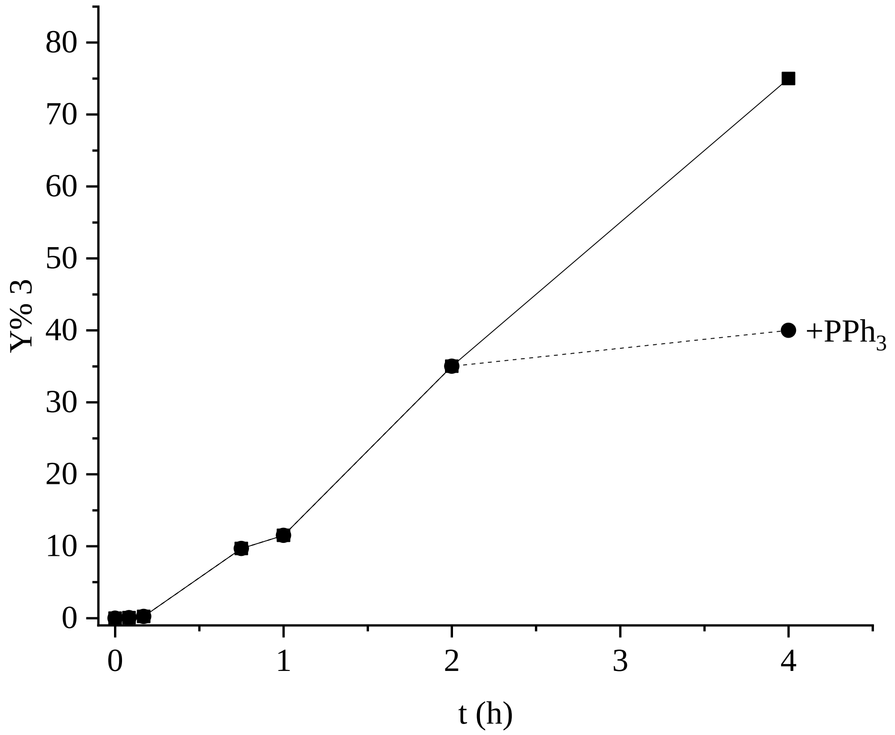

Figure S7. Quenching test with triphenylphosphine for the oxidation of neat benzyl alcohol **1a** to benzoic acid **2a** at 150 ºC and 4 bars of O_2, catalyzed by $Pd(OAc)_2$.

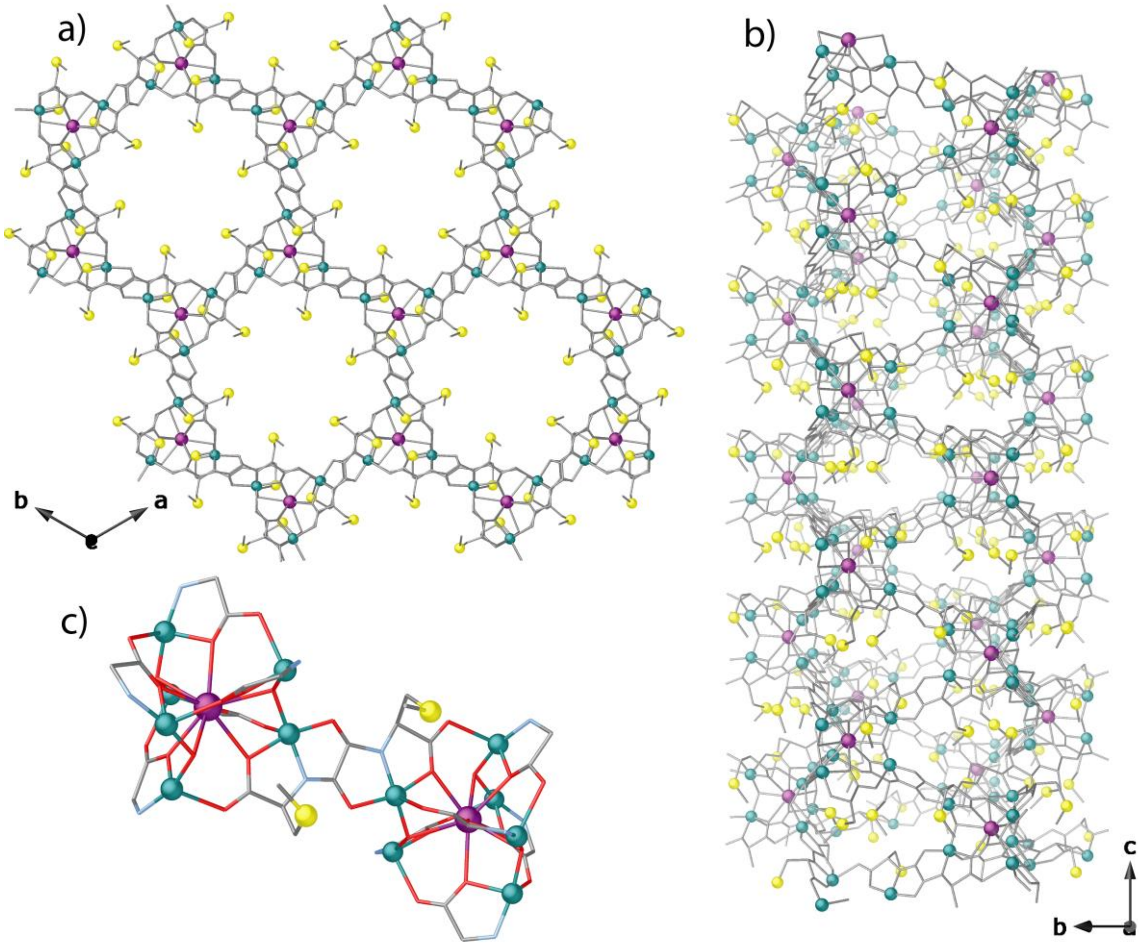

Figure S8. a) Views of a fragment of **3** in the *ab* and *bc* (c) planes, respectively. c) Fragment of **3** showing the dianionic bis(hydroxo) dicopper(II) building blocks. Copper, strontium and sulfur atoms are represented by green, purple and yellow spheres, respectively, whereas the ligands (except sulfur) are depicted as grey sticks [carbon: gray, oxygen: red and nitrogen: blue in fragment represented in c)]. Hydrogen atoms are omitted for clarity.

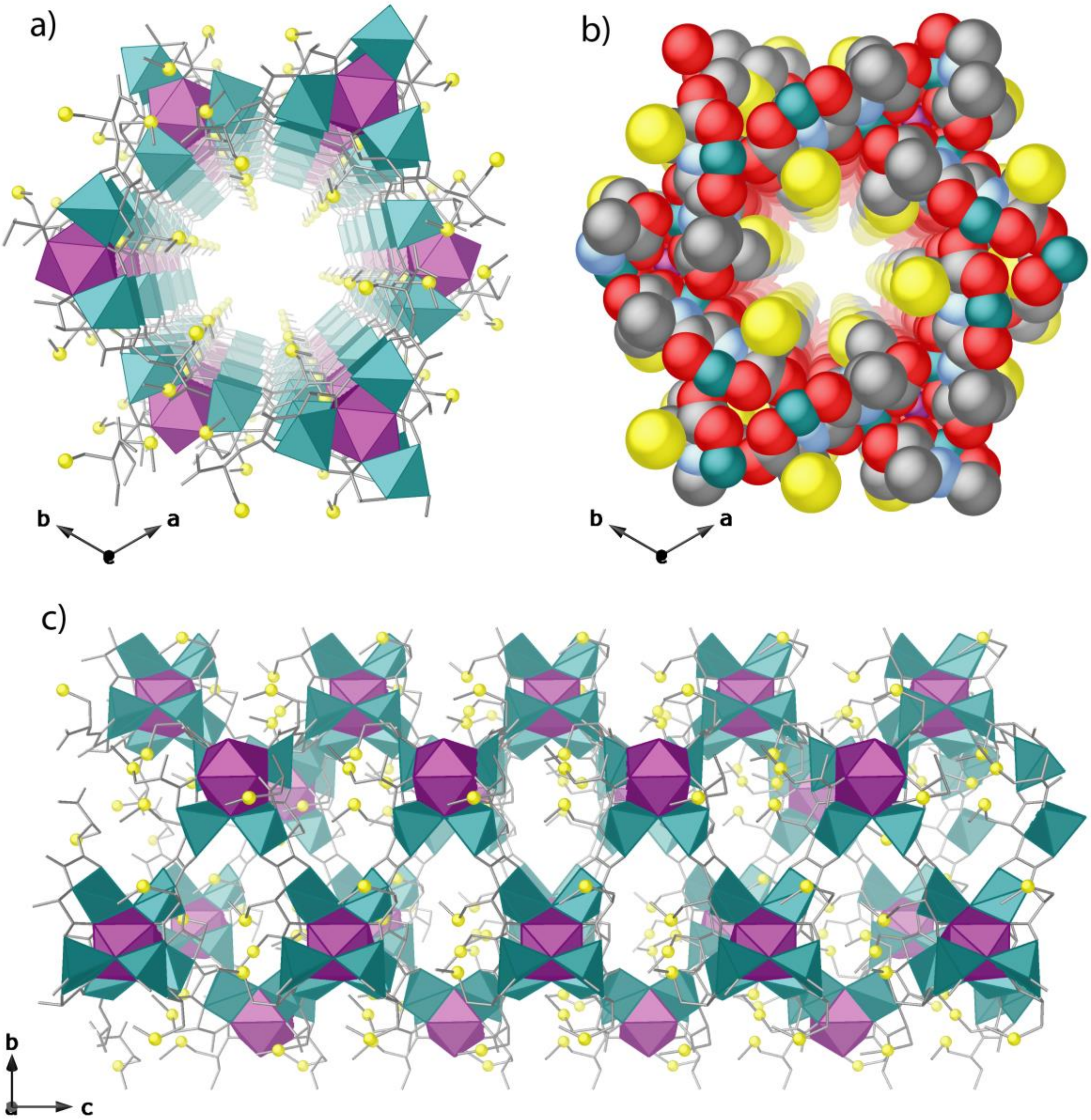

Figure S9. (a) Perspective views of one single channel of **3** along the *c* axis with polyhedral (a) and space-filling model (b). In c) has been depicted a polyhedral view of a single channel along axis, showing the [001] propagation direction. Copper and strontium atoms from the network are represented by cyan and purple polyhedra, respectively, whereas organic ligands are depicted as gray sticks. Yellow spheres represent S atoms. In the space-filling model (b) copper, strontium, carbon, oxygen, nitrogen and sulfur have been depicted as cyan, purple, gray, red, blue and yellow spheres, respectively.

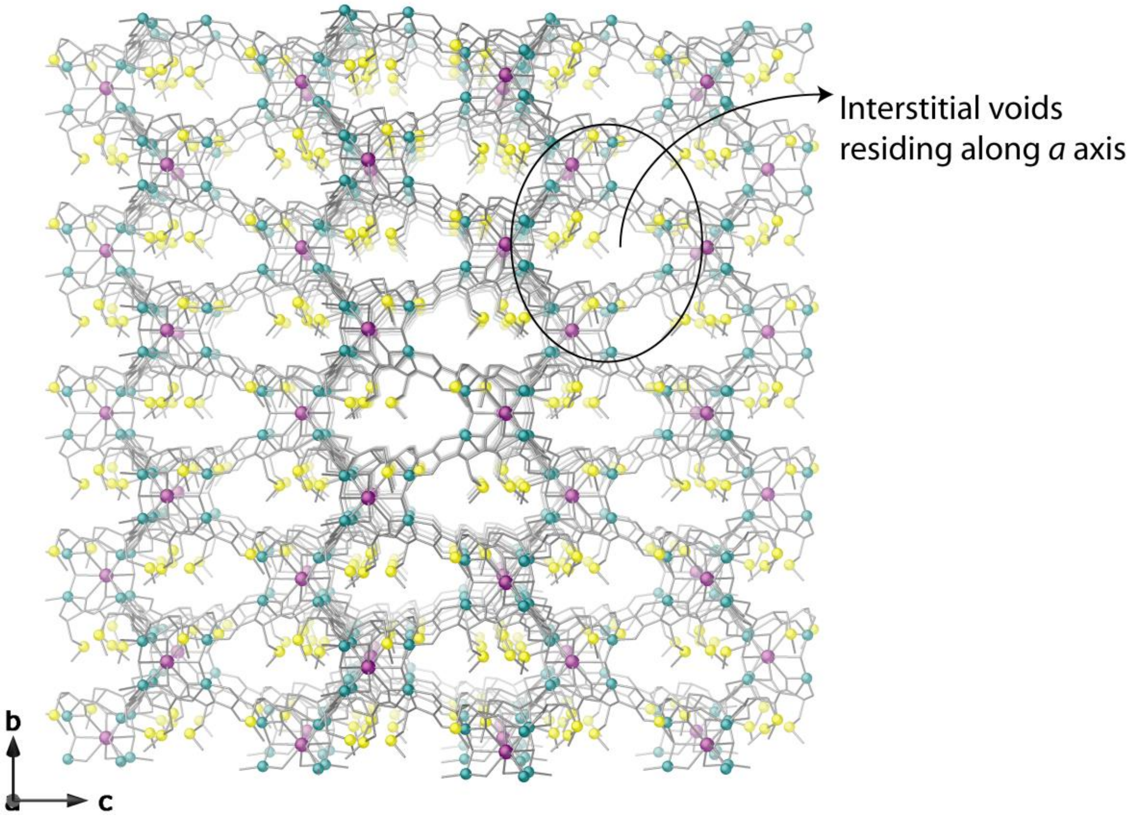

Figure S10. (a) Perspective view along *a* crystallographic axis of the 3D chiral porous network of **3** evidencing the regularly bent dymethyl thioether arm oft he mecysmox ligand with the terminal methyl groups pointing towards the smaller interstitial voids residing along *a* axis. Copper, strontium and sulfur atoms are represented by green, purple and yellow spheres, respectively, whereas the ligands (except sulfur) are depicted as grey sticks [carbon: gray, oxygen: red and nitrogen: blue in fragment represented in c)]. Hydrogen atoms are omitted for clarity.

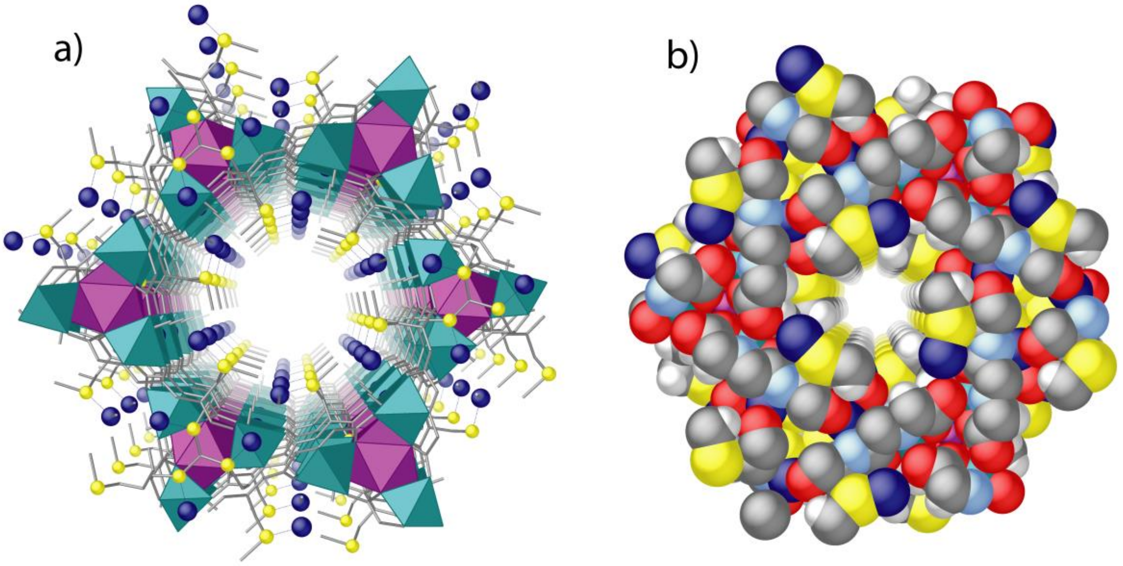

Figure S11. Details of pores in **4** viewed along *c* crystallographic axis in (a) polyhedral (copper and strontium) ball and stick for Pd^{2+} and sulfur ions/atoms, and stick (ligand) model or (b) space-filling model taking Van der Waals radii giving pore's dimensions of 0.7 nm. Copper and strontium atoms from the network are represented by cyan and purple polyhedra, respectively, whereas organic ligands are depicted as gray sticks. Yellow and blue spheres represent S and Pd atoms. In the space-filling model (b) copper, strontium, palladium, carbon, oxygen, nitrogen and sulfhur have been depicted as cyan, purple, deep blue, gray, red, light blue and yellow spheres, respectively. Little blue color sticks represent the Pd···S interactions.

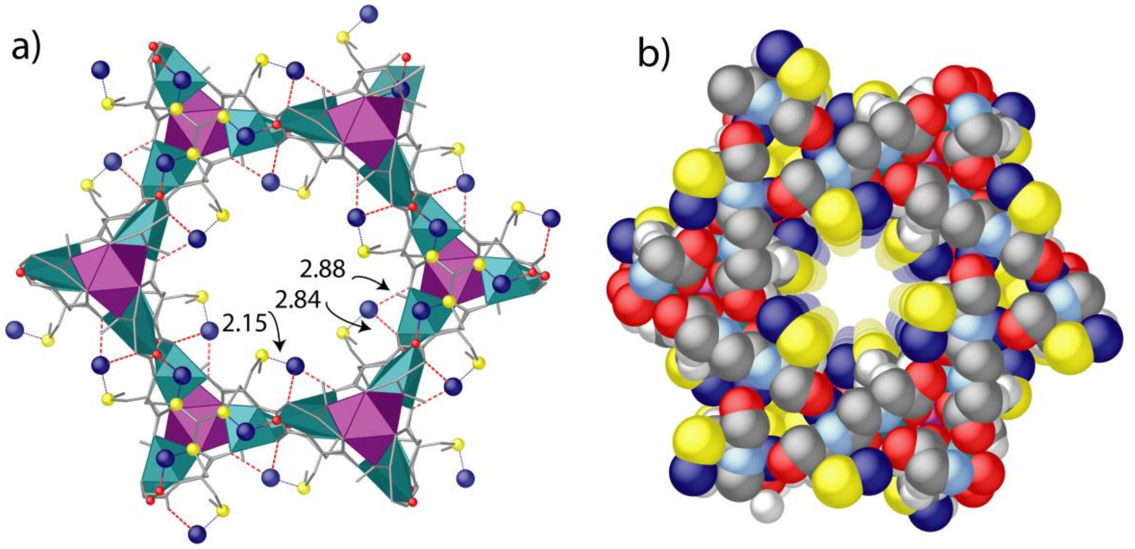

Figure S12. Details of pores in **5** viewed along *c* crystallographic axis in (a) polyhedral (copper and strontium) ball and stick for Pd^{2+} and Sulfur ions/atoms, and stick (ligand) model or (b) space-filling model taking Van der Waals radii giving pore's dimensions of 0.7 nm. Copper and strontium atoms from the network are represented by cyan and purple polyhedra, respectively, whereas organic ligands are depicted as gray sticks. Yellow and blue spheres represent S and Pd atoms. In the space-filling model (b) copper, strontium, palladium, carbon, oxygen, nitrogen and sulfur have been depicted as cyan, purple, deep blue, gray, red, light blue and yellow spheres, respectively. Blue dashed lines represent the Pd···S interactions whereas red dashes lines represent $Pd^{2+}\cdots O_{ligand}$ interactions.

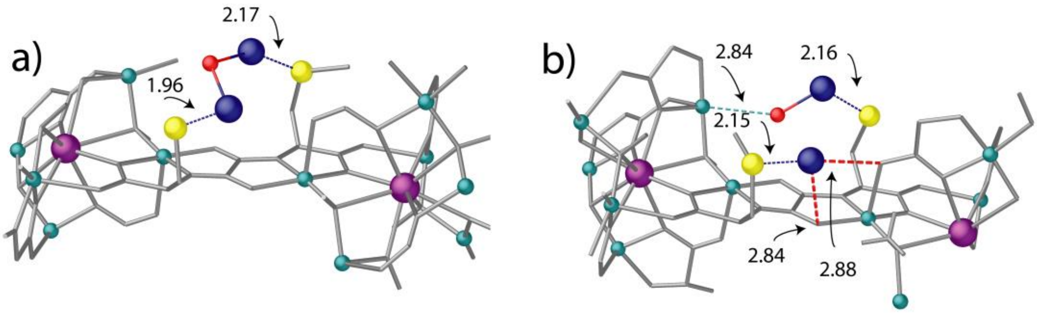

Figure S13. Details of the Pd···S interactions in **4** (a) and **5** (b) underlining the detected water molecule acting as bridge between two-coordinated Pd^{2+} ions in **4**, still remains coordinated in **5**, as terminal ligand, only to Pd^{2+} metal ions as a consequence of the breaking linkage after Pd^{0} SAC formation. Copper, strontium, palladium, oxygen and sulfur atoms are represented by green, purple, blue, red and yellow spheres, respectively, whereas the ligands (except sulfur) are depicted as grey sticks. Hydrogen atoms are omitted for clarity.

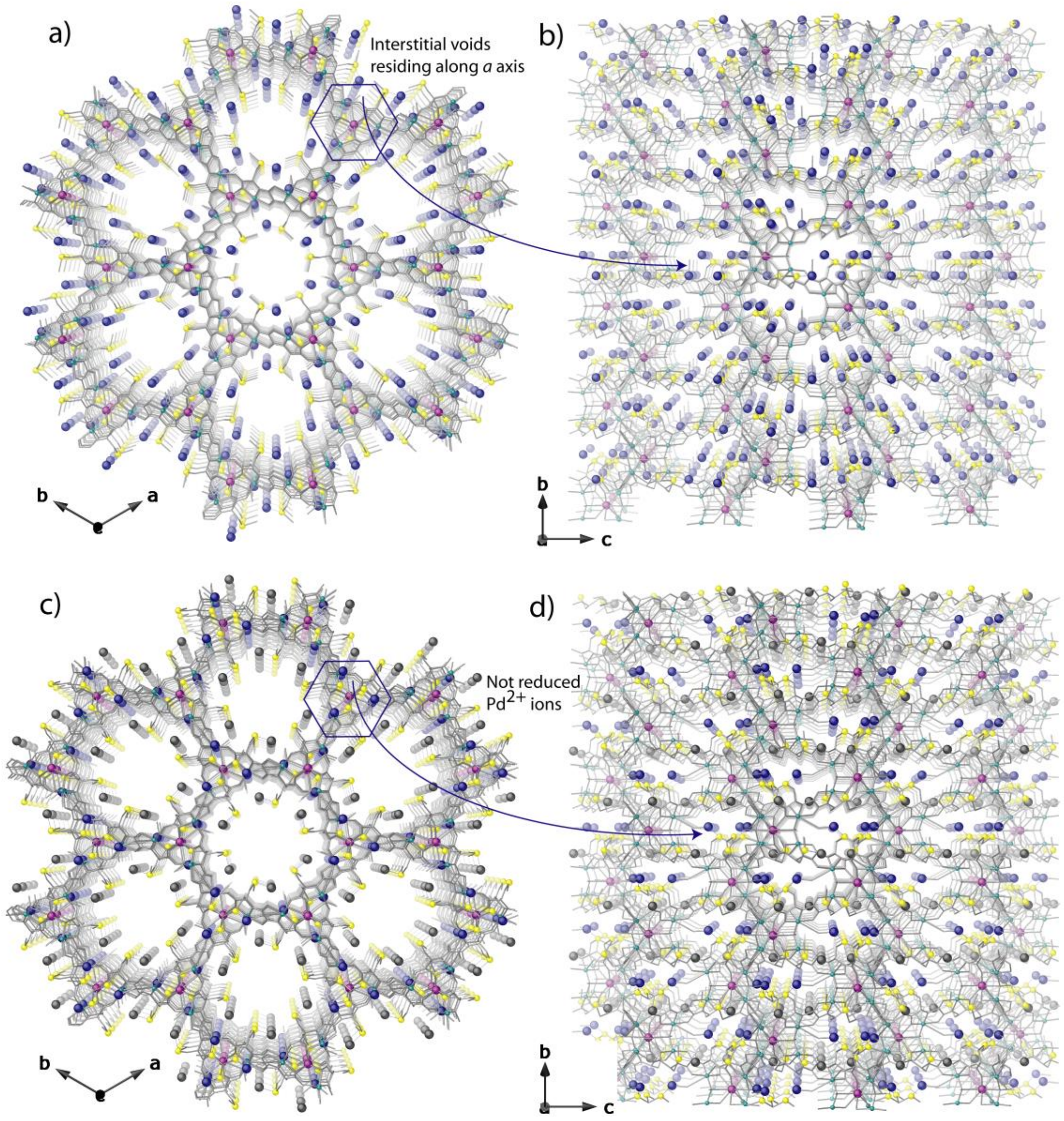

Figure S14. Details of fragments of the net along *c* and *a* crystallographic axis for **4** (a-b) and **5** (c-d) respectively, comparing the positions of Pd^{2+} ions residing both in big pores and smaller interstitial voids (**4**) and Pd^0 single atom catalyst (depicted as grey spheres) residing only in biggest pores in **5** where Pd^{2+} (depicted still as blue spheres) residing in smaller voids are still not reduced. Copper, strontium and sulfur atoms of the MOF are represented by green, purple and yellow spheres, respectively, whereas the ligands (except sulfur) are depicted as grey sticks [carbon: gray, oxygen: red and nitrogen: blue in fragment represented in c)]. Hydrogen atoms are omitted for clarity.

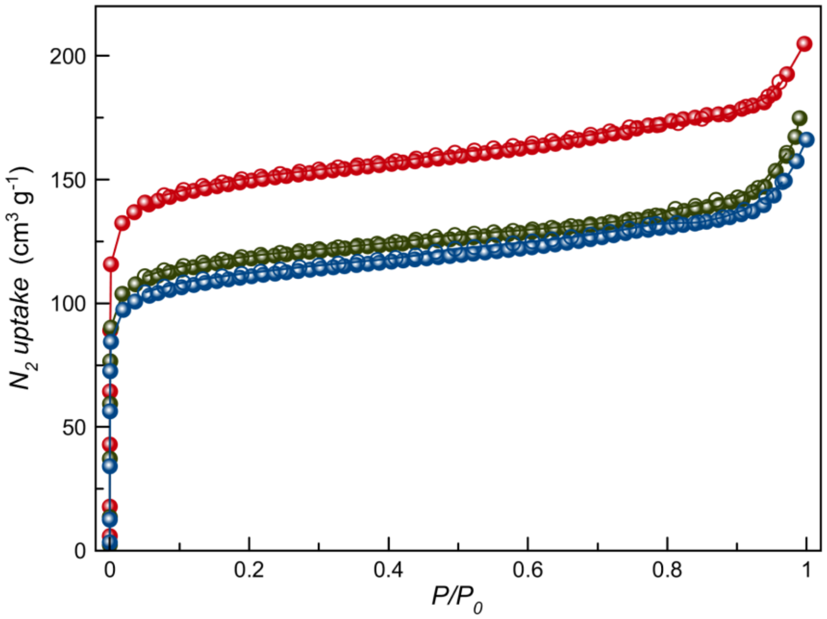

Figure S15. N_2 (77 K) adsorption isotherms for the activated compounds **3** (red), **4** (blue) and **5** (green). Filled and empty symbols indicate the adsorption and desorption isotherms, respectively. The samples were activated at 70 °C under reduced pressure for 16 h prior to carry out the sorption measurements.

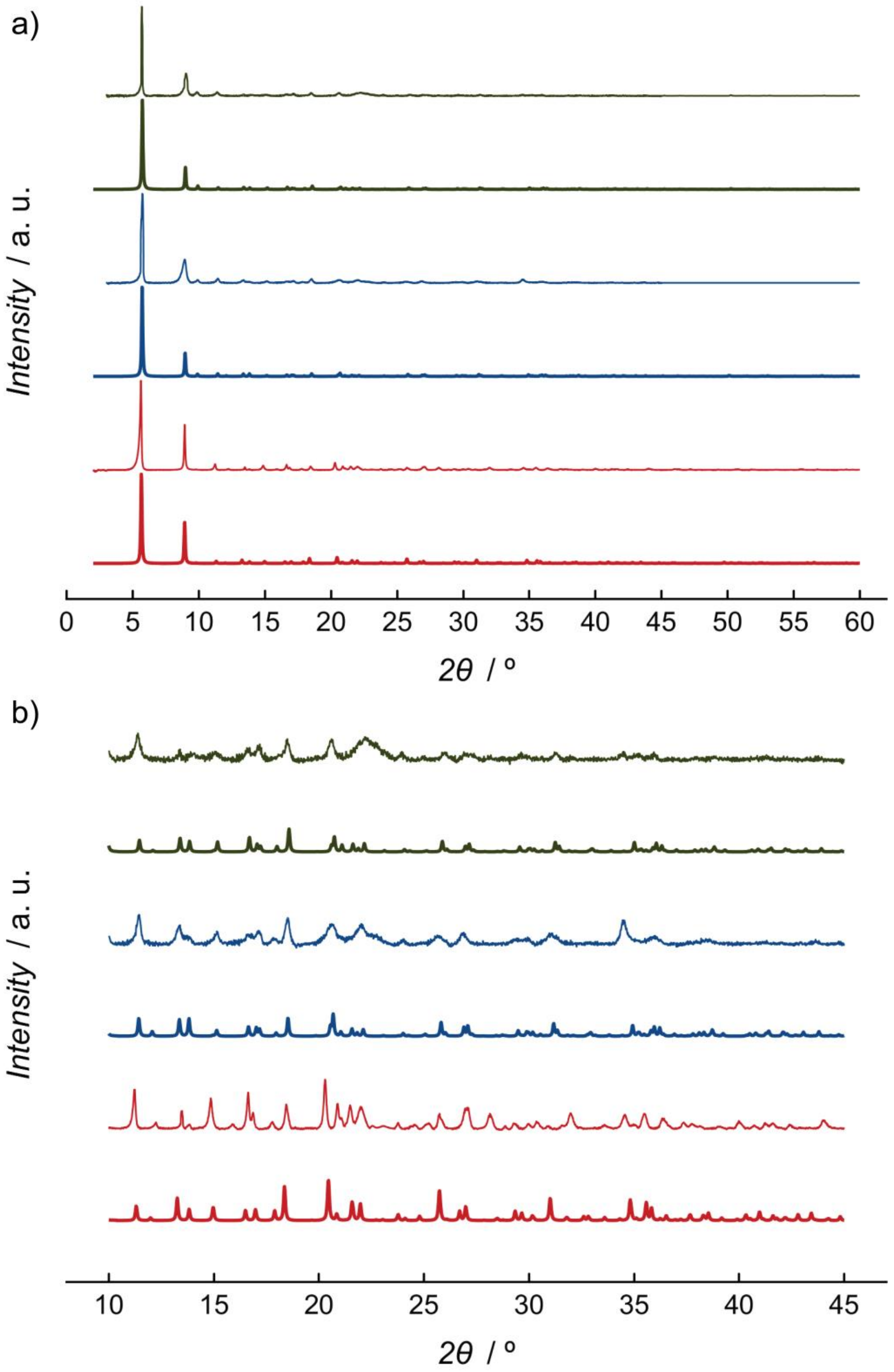

Figure S16. a) Calculated (bold lines) and experimental (solid lines) PXRD pattern profiles of **3** (red), **4** (blue) and **5** (green) in the 2θ range 2.0–60.0°. b) Calculated (bold lines) and experimental (solid lines) PXRD pattern profiles of **3** (red), **4** (blue) and **5** (green) are shown in the 2θ range 10.0–60.0° for the sake of clarity.

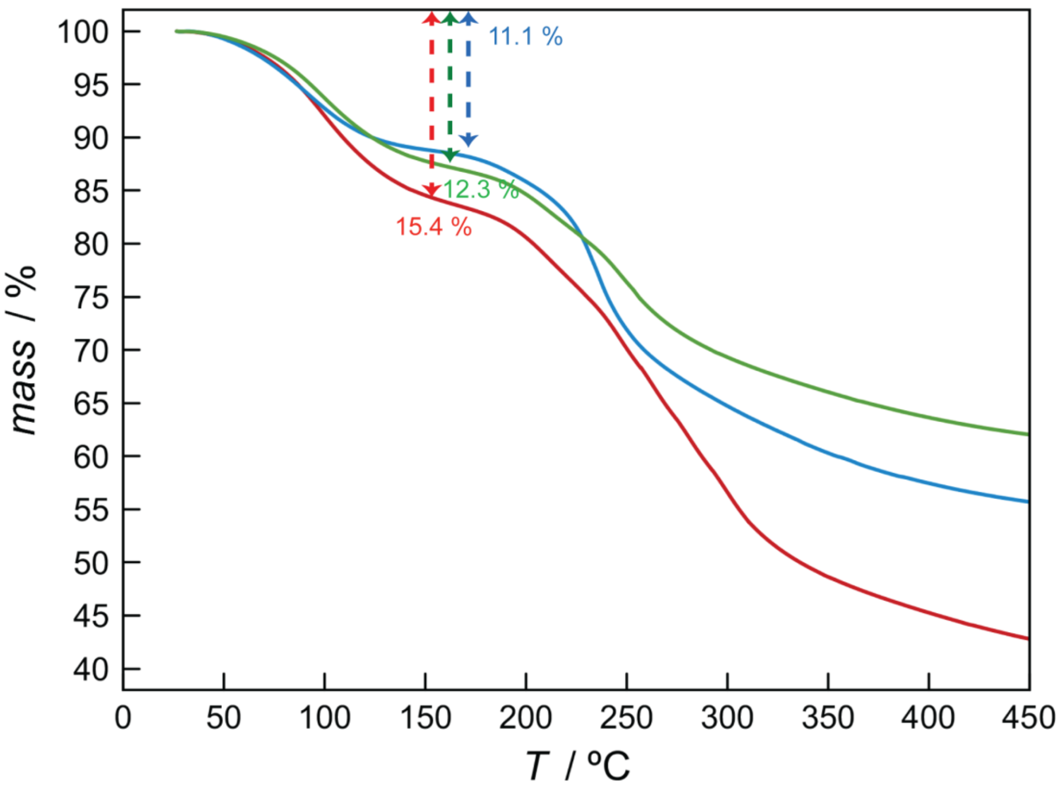

Figure S17. Thermo-Gravimetric Analysis (TGA) of **3** (red), **4** (blue) and **5** (green) under dry N_2 atmosphere.

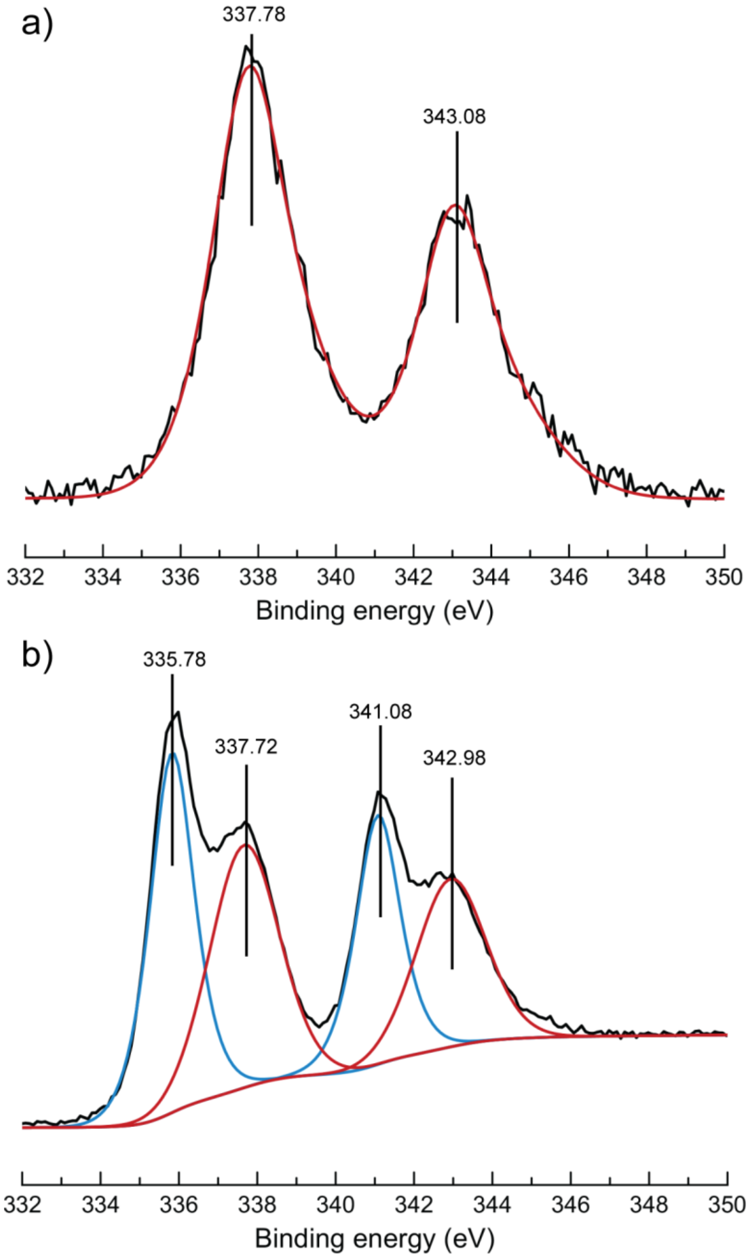

Figure S18. X–ray photoelectron spectroscopy (XPS) of **4** (a) and **5** (b).

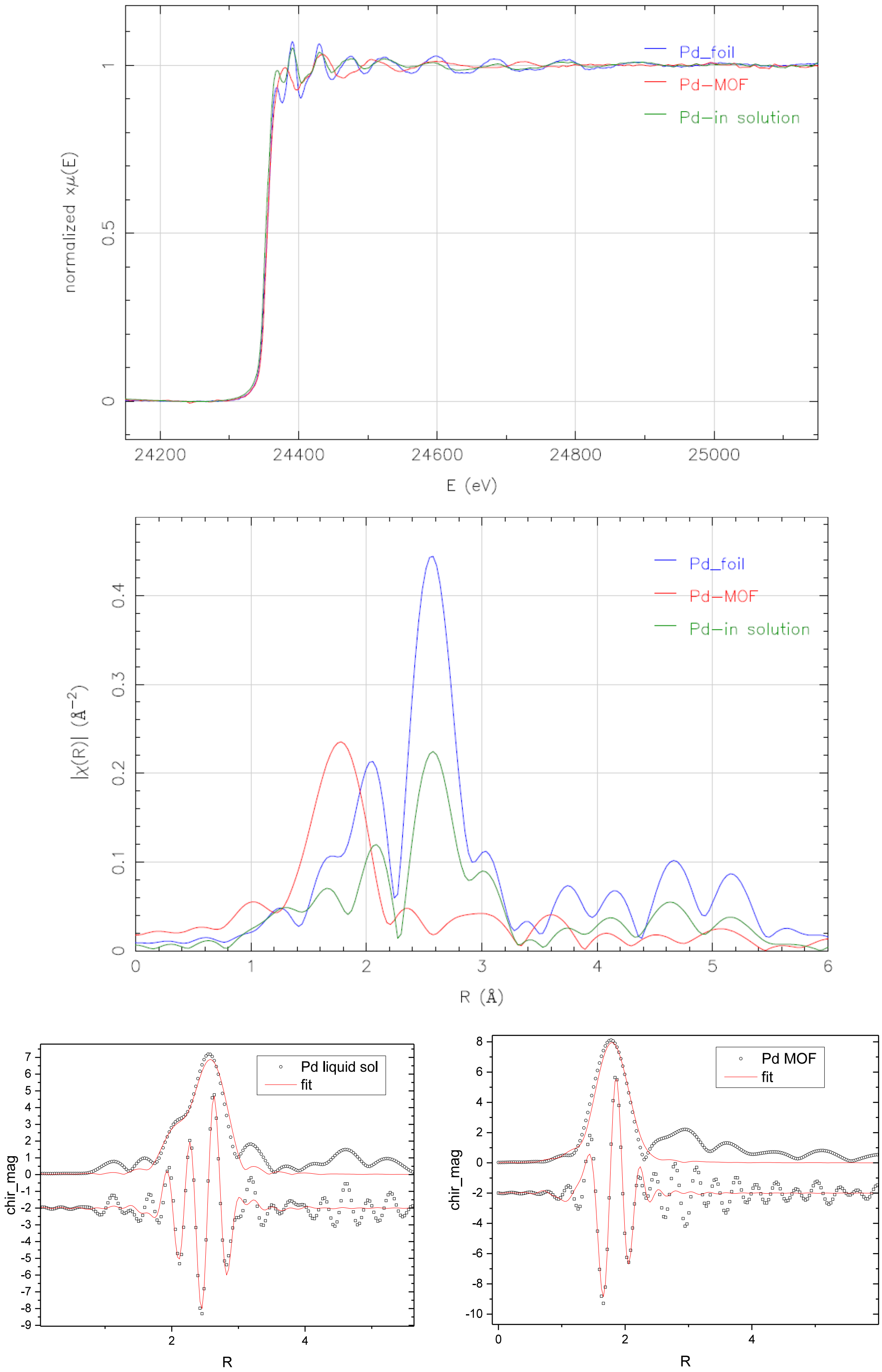

Figure S19. XANES (top) and EXAFS (middle) spectra of Pd in solution (green lines) and fresh MOF **5** (red lines), compared to Pd foil (blue lines). Fitting (bottom) for both Pd catalysts.

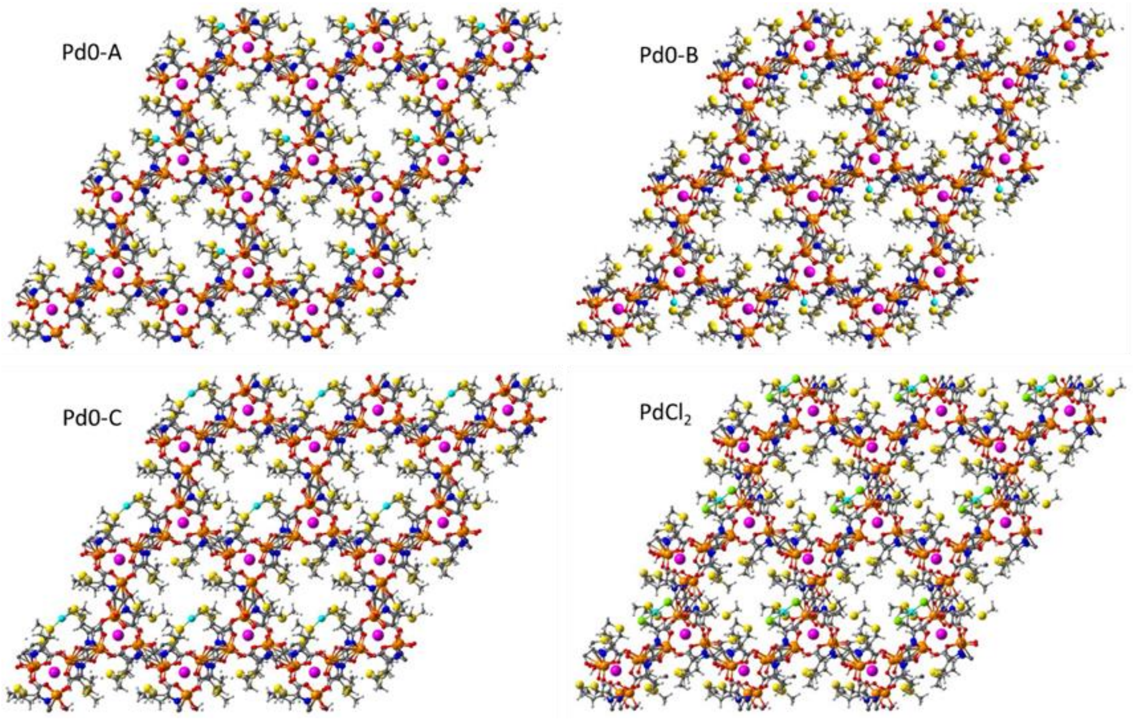

Figure S20. General view of the DFT optimized structures of Pd(0) and $PdCl_2$ within the MOF, for three different coordination environments (Pd0-A-C). Pd, Cu, Sr, S, N, O, Cl, C and H atoms are depicted as cyan, orange, pink, yellow, blue, red, green, grey and white balls, respectively.

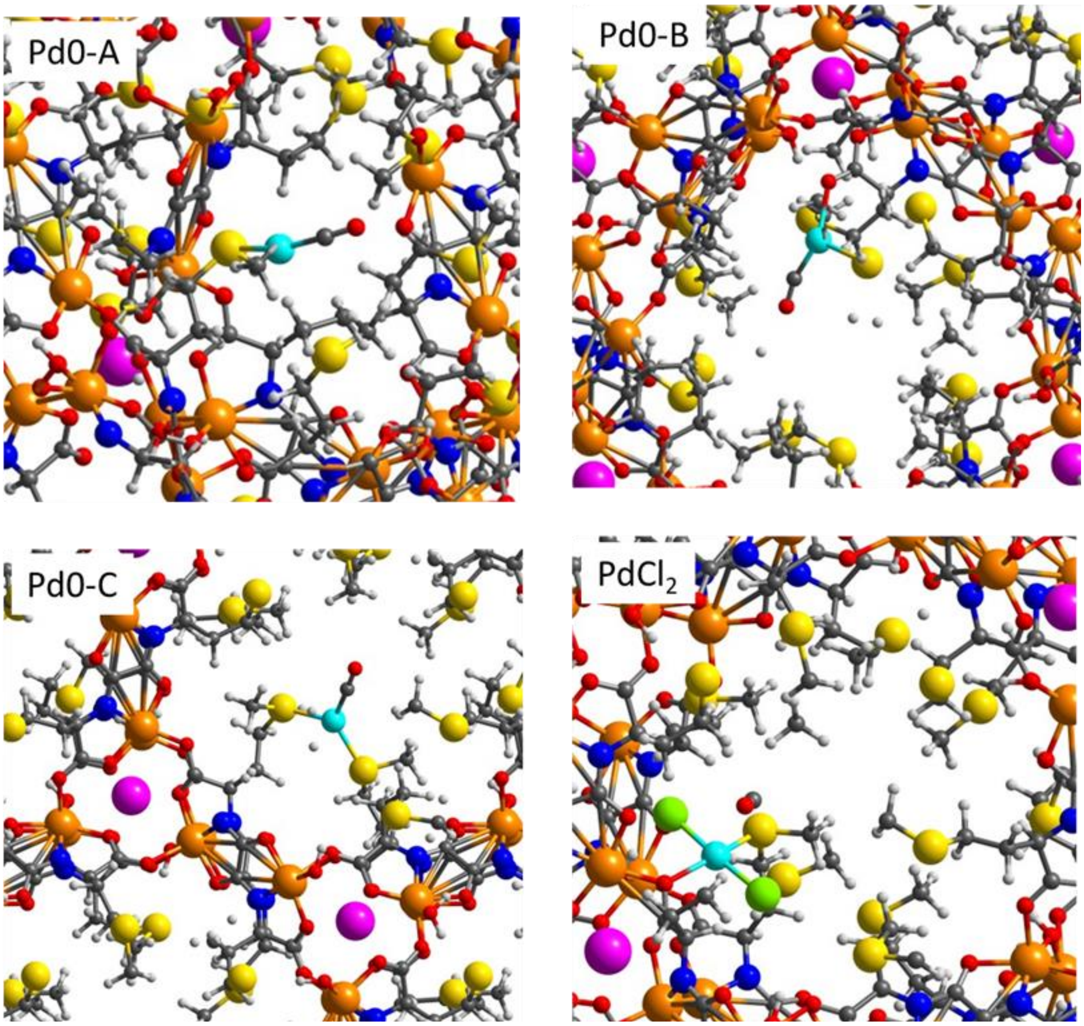

Figure S21. DFT optimized structures of CO interacting with Pd(0) and $PdCl_2$ within the MOF. Pd, Cu, Sr, S, N, O, Cl, C and H atoms are depicted as cyan, orange, pink, yellow, blue, red, green, grey and white balls, respectively.

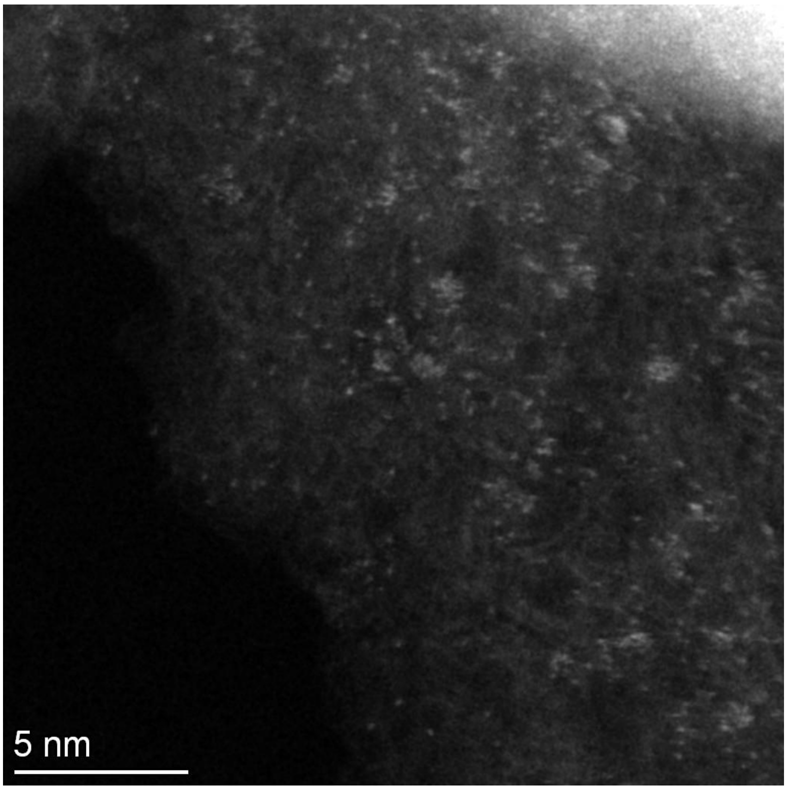

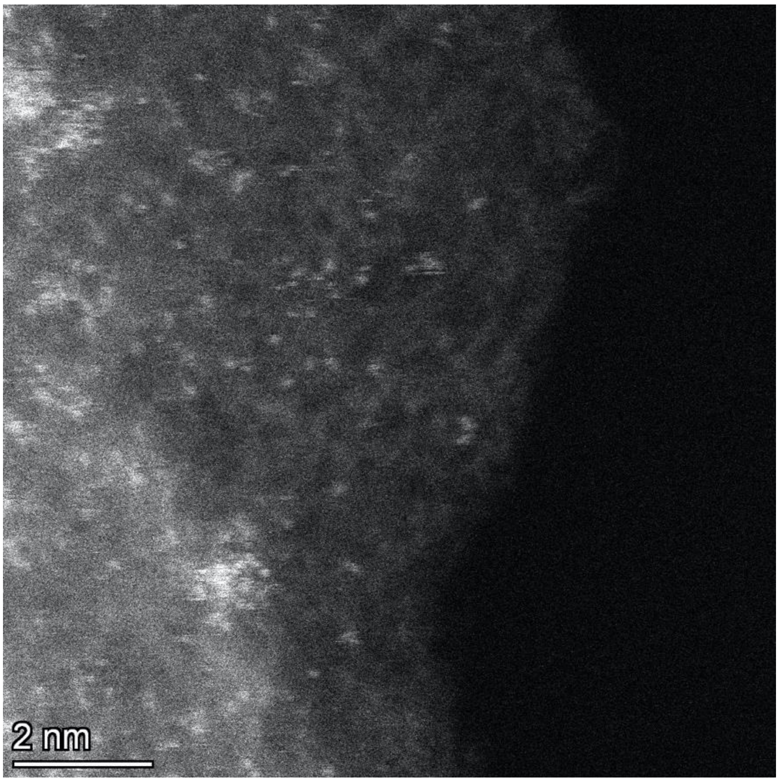

Figure S22. Representative AC-HAADF-STEM images of MOF **5** sample after catalytic experiments, at different magnifications, showing the presence of Pd SACs. The corresponding location of subnanometric Pt species are clearly visualized thanks to the higher atomic number (Z) in comparison to MOF chemical composition elements.

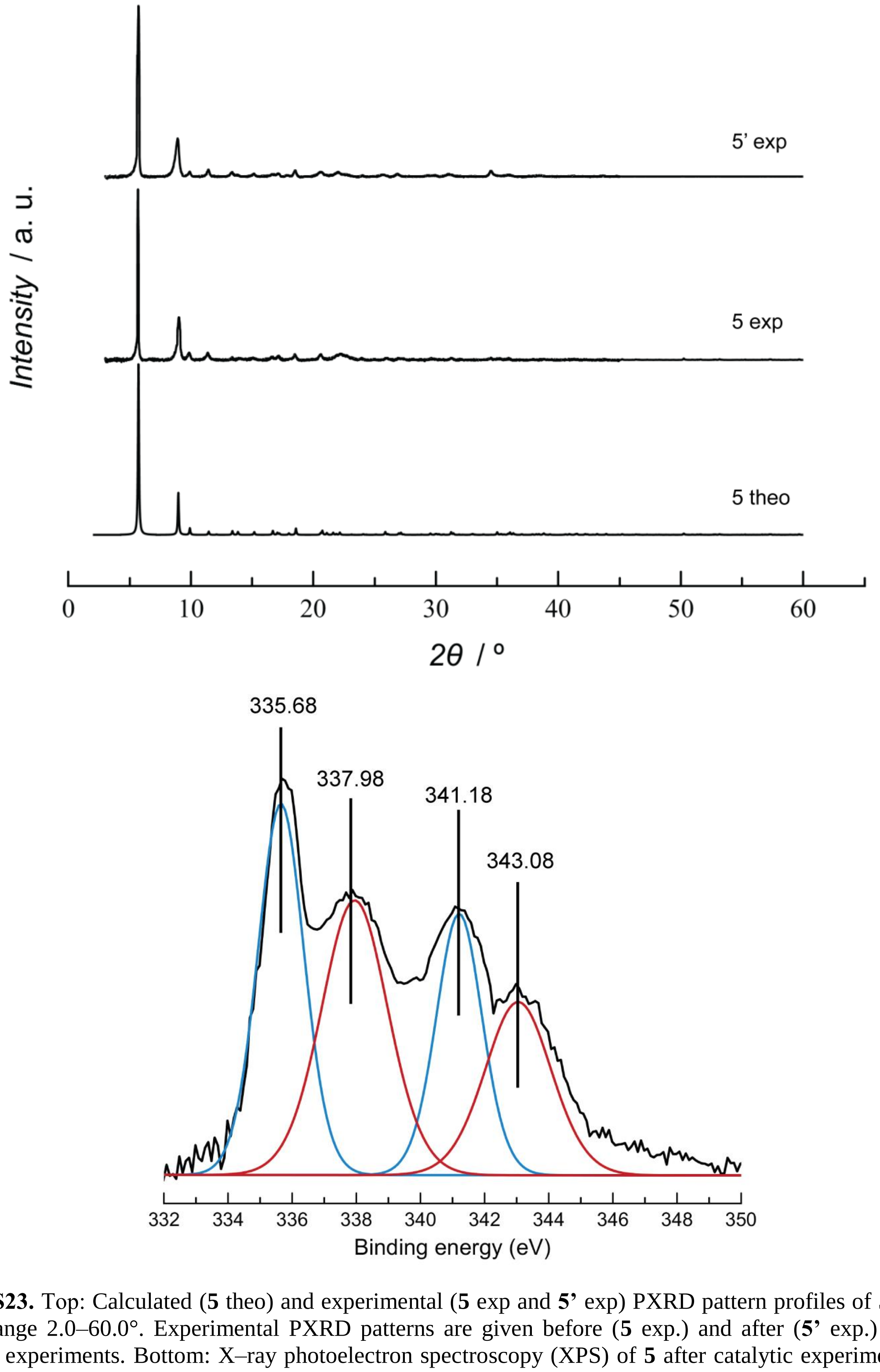

Figure S23. Top: Calculated (**5** theo) and experimental (**5** exp and **5’** exp) PXRD pattern profiles of **5** in the 2θ range 2.0–60.0°. Experimental PXRD patterns are given before (**5** exp.) and after (**5’** exp.) the catalytic experiments. Bottom: X–ray photoelectron spectroscopy (XPS) of **5** after catalytic experiments (**5‘**).

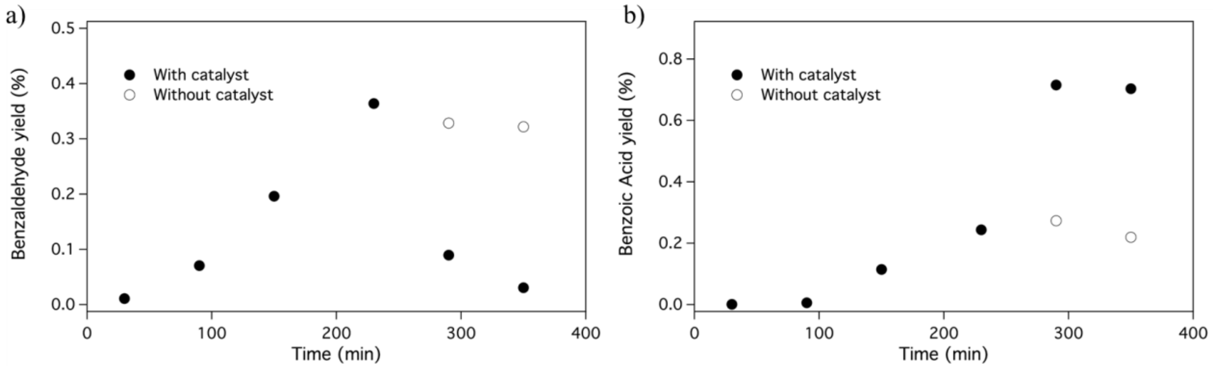

Figure S24. Hot filtration test (without catalyst refers to filtrated catalyst) for the aerobic oxidation of **1a** catalyzed by MOF **5** under optimized reaction conditions. Reaction conditions: 1.96 mmol substrate, Pd_1 SACs-MOF 0.3% mol [Pd], 4 atm O2, 150°C, 450 rpm. GC yields.

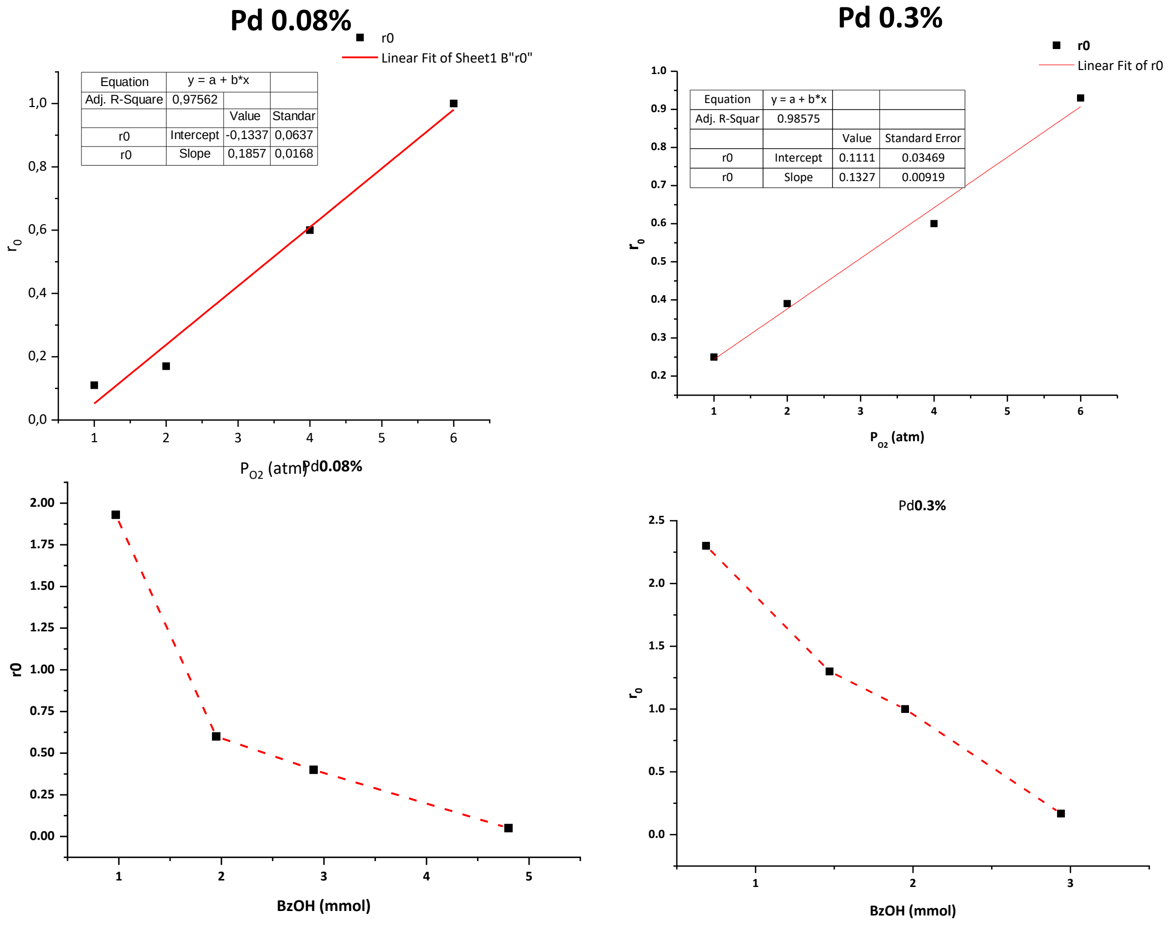

Figure S25. Initial rate-concentration plots for the reaction. Reaction orders for O_2 and benzyl alcohol **1a** are similar (+1 and -1, respectively) at different Pd_1 loadings, at 150 ºC (compare left and right graphs).

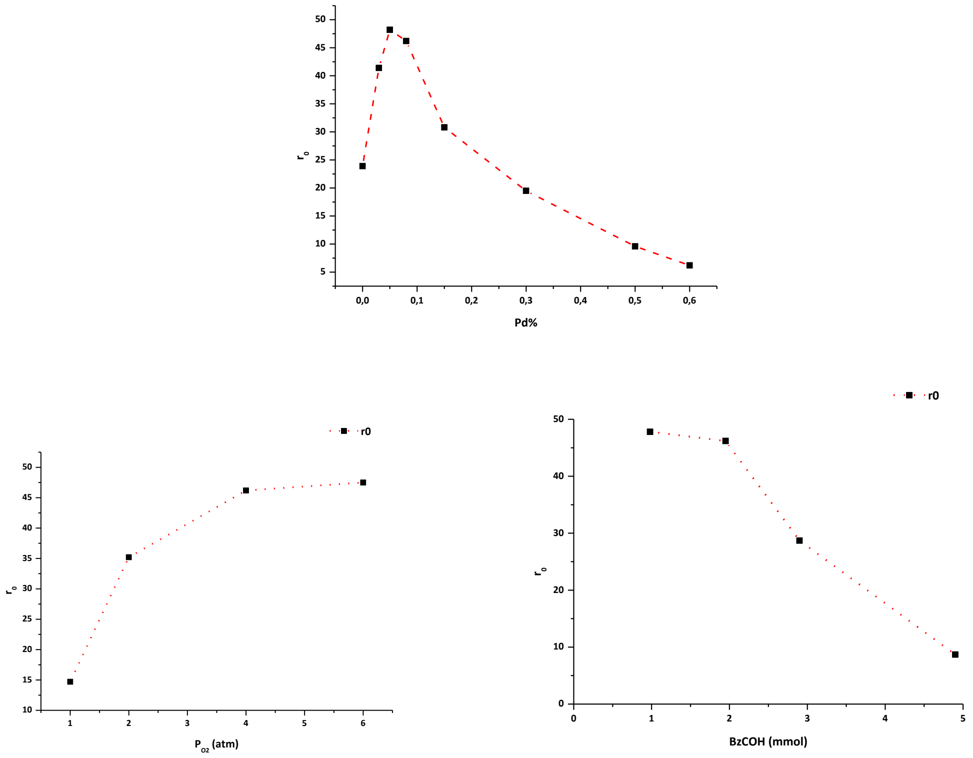

Figure S26. Initial rate-concentration plots when starting from neat benzaldehyde instead of benzyl alcohol **1a**, at 150 ºC. Reaction orders are similar to when starting from benzyl alcohol **1a**.

a)

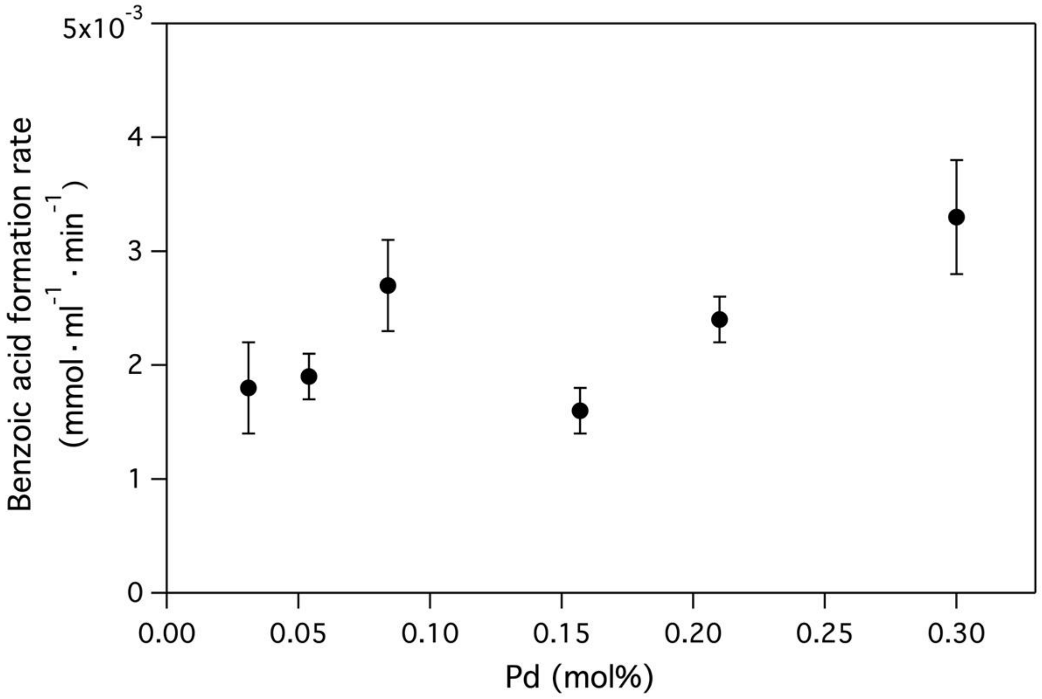

b)

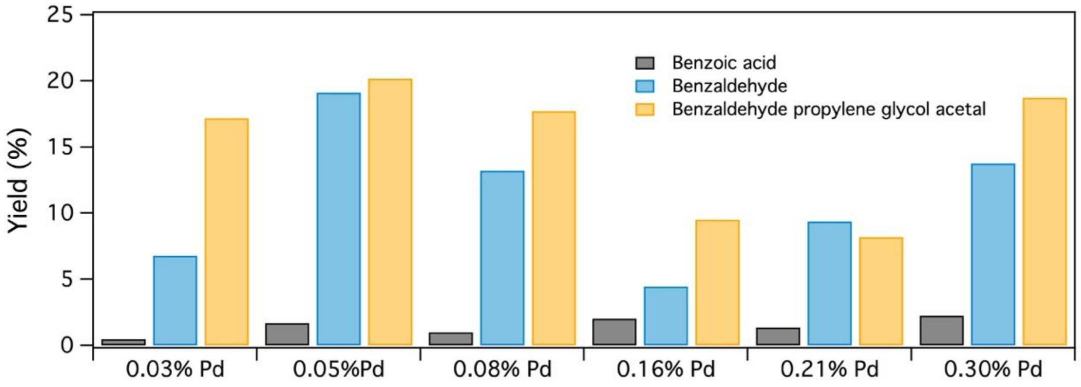

Figure S27. a) Initial rates for benzoic acid **2a** formation in presence of propylene glycol. The preferred formation of the acetal respect to **2a** lowers the initial rate. The formation rate of **2a** decreased by a factor of approximately 4 to 16, with a sharper decrease at higher Pd concentrations. Error bars account for a standard deviation of approximately 5%. b) Yields of the reactions after 40-60 min.

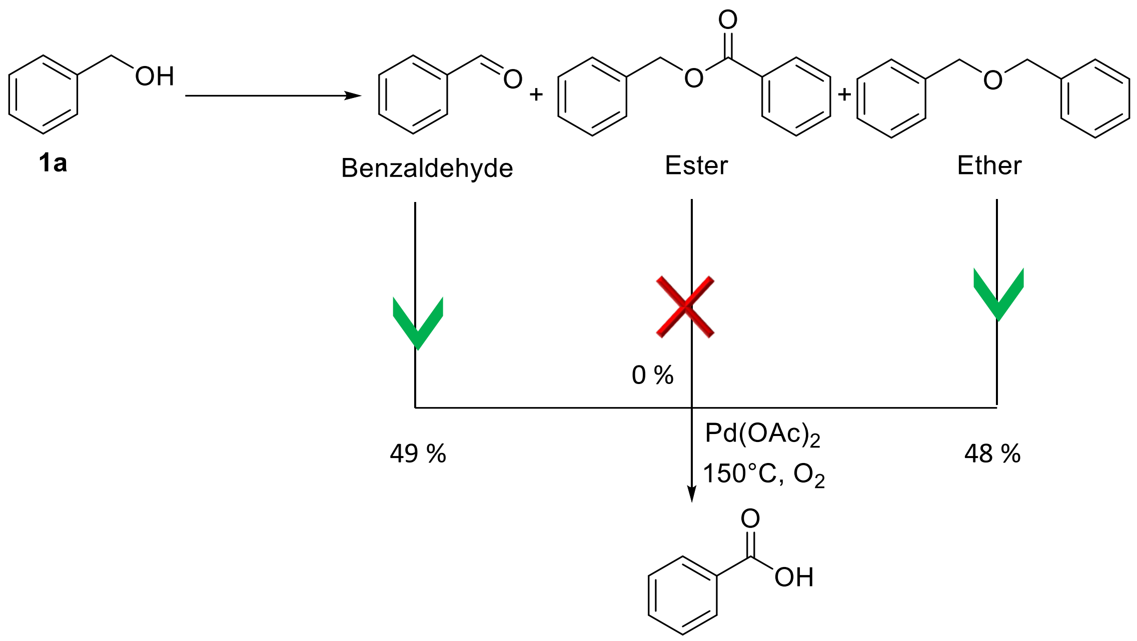

Figure S28. Study of benzoic acid **2a** formation from benzaldehyde, the corresponding ester and dibenzyl ether.

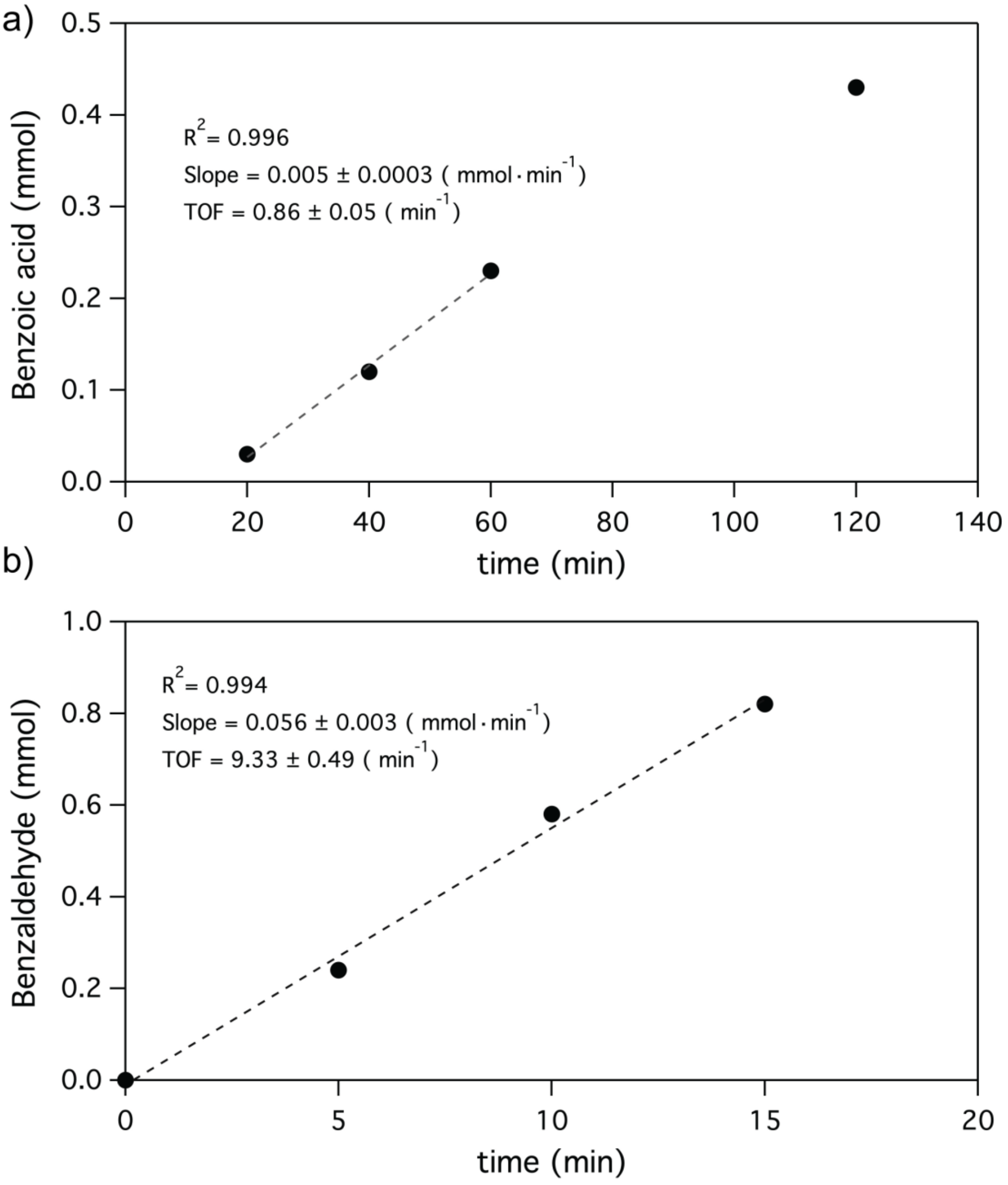

Figure S29. Radical inhibitor (DABCO) experiment, showing the initial rates for the formation of a) benzoic acid **2a** and b) benzaldehyde. The total conversion of the alcohol to aldehyde is achieved at a similar rate than under normal conditions, while the rate of formation of benzoic acid was approximately 5 times lower. Reaction conditions: 2 mmol of benzyl alcohol, 0.3% mol $Pd(OAc)_2$, 150 ºC, 4 bar O_2.

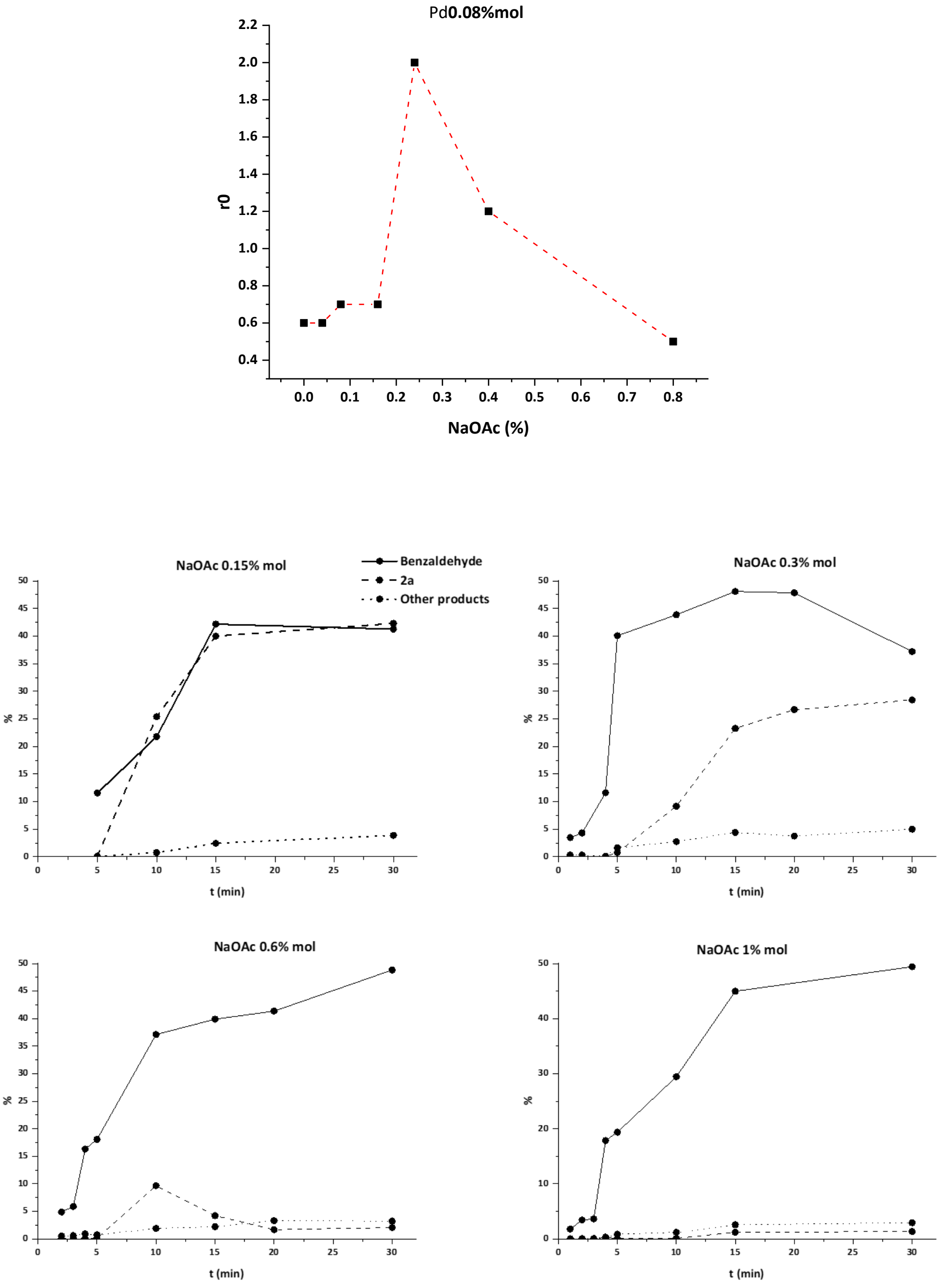

Figure S30. Variation of the reaction rate with NaOAc for 0.08 mol% of $Pd(OAc)_2$ and kinetics for 0.3 mol% of $Pd(OAc)_2$.

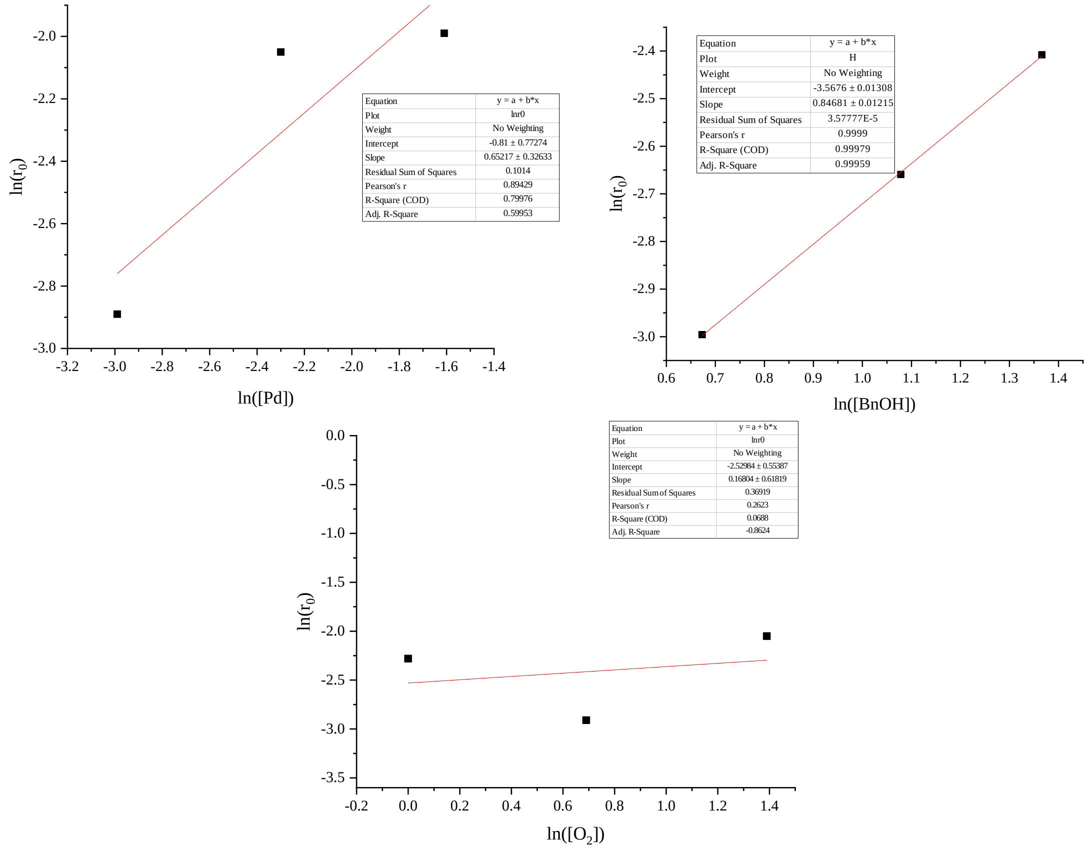

Figure S31. Initial rate-concentration plots for the reaction catalyzed by MOF **5**. Reaction orders for MOF **5** and benzyl alcohol **1a** are similar (+1), while the reaction order for O_2 is likely 0.

References

1. SAINT, version 6.45, Bruker Analytical X-ray Systems, Madison, WI, 2003.

2. Sheldrick G.M. SADABS Program for Absorption Correction, version 2.10, Analytical X-ray Systems, Madison, WI, 2003.

3. (a) Evans, P. Sclaing and assessment of data quality. *Acta Cryst. D* **62**, 72 (2006). (b) Evans, P. R., Murshudov, G. N. How good are my data and what is the resolution?. *Acta Cryst. D* **69**, 1204 (2013). (c) Winn, M. D. *et al*. Overview of the *CCP4* suite and current developments. *Acta Cryst. D* **67**, 235 (2011). (d) Winter, G. *xia2*: and expert system for macromolecular crystallography data reduction. *J. Appl. Cryst.* **43**, 186 (2010). (e) Winter, G. *et al*. *DIALS*: implementation and evaluation of a new integration package *Acta Cryst*. **2018**, D74, 85-97

4. (a) Sheldrick, G. M. Crystal structure refinement with SHELXL. *Acta Cryst. C* **71**, 3 (2015). (b) Sheldrick, G. M. A short history of SHELX. *Acta Cryst. A* **64**, 112 (2008). (c) SHELXTL-2013/4, Bruker Analytical X-ray Instruments, Madison, WI, 2013.

5. (a) Spek, A. L. *PLATON* SQUEEZE: a tool for the calculation of the disordered solvent contribution to the calculated structure factors. *Acta Crystallogr. Sect. C-Struct. Chem*. **71**, 9 (2015). (b) Spek, A. L. Structure validation in chemical crystallography. *Acta Crystallogr. Sect. D, Biol. Crystallogr.* **65**, 148 (2009).

6. Farrugia, L. J. *WinGX* suite for small-molecule single-crystal crystallography. *J. Appl. Crystallogr.* **32**, 837 (1999).

7. Palmer, D. CRYSTAL MAKER, Cambridge University Technical Services, C. No Title, 1996.

8. Kresse, G.; Furthmüller, Efficient iterative schemes for *ab initio* total-energy calculations using a plane-wave basis set, *J. Phys. Rev. B* **54**, 11169 (1996).

9. a) Perdew, J. P.; Burke, K.; Ernzerhof, M. Generalized Gradient Approximation Made Simple. *Phys. Rev. Lett.* **77**, 3865 (1996). b) Perdew, J. P.; Burke, K.; Ernzerhof, M. Generalized Gradient Approximation Made Simple. *Phys. Rev. Lett.* **78**, 1396 (1997).

10. Blöchl, P. E. Projector augmented-wave method. *Phys. Rev. B* **50**, 17953 (1994).

11. a) Henkelman, G.; Arnaldsson, A.; Jonsson, H. A fast and robust algorithm for Bader decomposition of charge density. *Comput. Mater. Sci.* **36**, 354 (2006). b) Sanville, E.; Kenny, S. D.; Smith, R.; Henkelman, G. Improved Grid-Based Algorithm for Bader Charge Allocation. *J. Comput. Chem.* **28**, 899 (2007).

12. Simonelli, L. et al. CLÆSS: The hard X-ray absorption beamline of the ALBA CELLS synchrotron. *Cog. Phys.* **3**, 1231987 (2016).

13. Ravel, B.; Newville, M. ATHENA, ARTEMIS, HEPHAESTUS: data analysis for X-ray absorption spectroscopy using IFEFFIT, *J. Synchr. Rad.* **12**, 537 (2005).

14. a) Pd-S: Grønvold, F.; Røst., E. The crystal structure of PdSe2 and PdS2. *Acta Cryst.* **10**, 329 (1957). b) Pd-Pd: Whyckoff R. W. G. Crystal Structures **1**, 7 (1963) Second edition. Interscience Publishers, New York, New York.
